# Colorimetric Sensor for Cr(VI) Ion Detection in Tap Water Using a Combination of AuNPs and AgNPs

**DOI:** 10.1021/acsomega.4c02699

**Published:** 2024-06-06

**Authors:** Kullavadee Karn-orachai, Panwadee Wattanasin, Aroonsri Ngamaroonchote

**Affiliations:** † National Nanotechnology Center (NANOTEC), National Science and Technology Development Agency (NSTDA), Klongluang 12120, Pathum Thani, Thailand; ‡ Faculty of Science, Center of Excellence for Innovation in Chemistry, Prince of Songkla University, Hat Yai 90110, Thailand

## Abstract

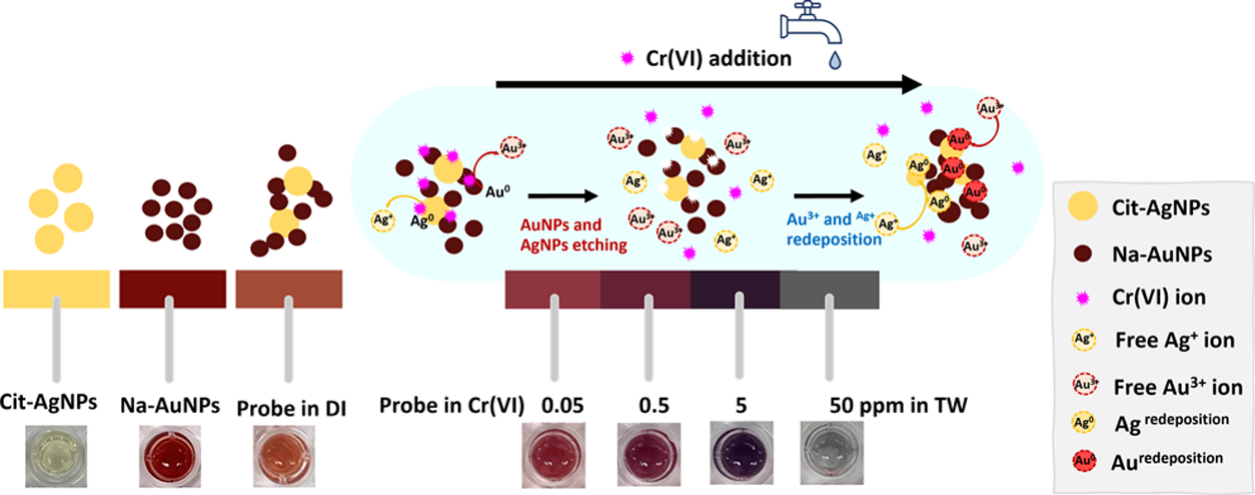

Colorimetric sensors are a promising technique for the simple screening of water, food, and environmental samples contaminated with interferents, allowing for color changes to be observed with the naked eye or a spectrophotometer. In this study, a colorimetric sensor for the selective detection of hexavalent chromium ion (Cr(VI)) contamination in water was developed. A combination of sodium borohydride-coated gold and citrate-capped silver nanoparticles (Na-AuNPs/cit-AgNPs) was employed as a colorimetric probe. Upon the addition of Cr(VI)-contaminated tap water in the colorimetric probe solution, the color sequentially transitioned from its initial orange to dark reddish-purple, dark purplish-red, dark blue-violet, and gray. This colorimetric strategy relies on AgNP dissolution and AuNP aggregation in the presence of the Cr(VI) ions. The dissolution of AgNPs is evidenced by the reduction of the characteristic peak of AgNPs at 400 nm, while the aggregation of AuNPs leads to a red shift in the absorption band at 514 nm, accompanied by broad absorption in the 500–700 nm range. The limits of detections were found to be 22.9 and 50 ppb using a spectrometer and by visual observation, respectively. The synthesized AuNPs and AgNPs are very stable in the presence of media containing complicated ions. We further demonstrated the practical applicability of the developed system for detecting Cr(VI) in real samples, including natural water and artificial urine, highlighting its potential for addressing Cr(VI) contamination in practical scenarios.

## 1 Introduction

Heavy-metal contamination has become a topic of focus for both domestic and international concerns regarding drinking water, natural water, tap water, herbs, cosmetic products, etc. Chromium is a heavy metal (HM) extensively utilized in numerous industries, such as dying, tanning, and printing ink, as well as in the production of magnetic tapes, batteries, and the plating industry.^1−4^ Cr can exist in two oxidation states: trivalent chromium (Cr(III)) and hexavalent chromium (Cr(VI)).^5^ Cr(III) is a vital mineral for both humans and other living organisms, while Cr(VI) is harmful.^5^ A small quantity of Cr(III) is not toxic to cells, whereas the accumulation of a large amount of Cr(III) can bind to the DNA structure and cause damage to cells.^5^ Short- and long-term Cr(VI) uptake is harmful to human health, causing nausea, stomach ulcers, skin ulcers, allergic reactions, kidney and liver damage, and lung and nasal cancers.^6^ It is reported that Cr(VI) is more toxic than Cr(III) due to its high solubility in water.^7−9^ Elevating the pH of a solution triggers the reduction of Cr(VI) to the nontoxic form Cr(III).^10^ Cr(VI) can easily contaminate water by forming various highly soluble complexes including chromic acid, chromates, polychromates, and dichromates, which elevate the problem of environmental water pollution.^11^ The World Health Organization (WHO) and Environmental Protection Agency (EPA) reported the maximum contamination level of Cr(VI) at about 0.01 ppm (0.2 μM) in drinking water.^12^ Moreover, the acceptable total chromium contents in drinking water issued by WHO and EPA were about 0.1 and 0.05 ppm, respectively.^3^ Therefore, it is very important to determine the amount of Cr in water before daily consumption.

Various analytical methods were employed for both direct and indirect determination of Cr, including atomic absorption spectroscopy,^13^ ion chromatography,^14^ inductively coupled plasma-atomic emission spectroscopy,^15^ fluorescent spectroscopy,^16^ electrochemistry,^17^ and UV–vis spectroscopy.^18^ These techniques show good performance for the highly sensitive detection of trace Cr with good accuracy and precision. However, there are some limitations, including expensive instruments, many sample preparation steps, the requirement of skilled personnel for operation, and a time-consuming process.^13−18^ Therefore, the development of a simple method for the selective determination of Cr(VI) in tap water becomes highly imperative.

Recently, noble metallic NPs, including gold, silver, Au@Ag, or Ag@Au core–shell nanostructures, have been widely employed as colorimetric probes for HM detection, which provide a higher extinction coefficient than organic dyes and good surface plasmon resonance properties.^19^ The color change after reaction can be recognized by both the naked eye and a spectrometer, which facilitates simple, sensitive, and selective HM detection.^19^ Numerous studies have reported the development of a colorimetric sensor for detecting Cr ions, which is based on the color change of a metallic nanoparticle (NP) colloid through two mechanisms, including NP aggregation/dispersion and etching mechanisms.^20−26^ The NP aggregation/dispersion is a simple mechanism based on the reduction of the interparticle distance among NPs after reaction with analytes, resulting in the formation of aggregates. The tendency of a change in the localized surface plasmon resonance (LSPR) spectra and transformation of the color of the colloidal solution during the reaction can be used to quantify the amount of analyte.^20−23^ Some NP aggregation/dispersion-based colorimetric sensors were developed by using various designs of noble metal NPs coated with different capping agents for the selective detection of Cr(VI), such as citrate-capped 45 nm AuNPs,^20^ gallic acid-capped AgNPs,^21^ and maleic acid-coated AuNPs.^22^ This approach would require highly stable NPs to eliminate autoaggregation, which directly influences the reliability of the sensor. In addition, the etching mechanism of metallic NPs relies on the particle dissolution induced by the analyte. This mechanism occurs when the standard reduction potential of the analyte is higher than that of NPs. The redox reaction between NPs and the HM ion leads to a change in the LSPR of NPs and colloidal solution color. The recent literature has reported strategies for developing redox etching-based colorimetric sensors for Cr(VI) determination, such as starch-coated AgNPs in the presence of ethylene diamine tetraacetic acid (EDTA) and sulfuric acid (H_2_SO_4_),^23^ Cu@Au core–shell NPs in the presence of hydrogen bromide (HBr),^24^ Ag@Au core–shell NPs in the presence of cetyltrimethylammonium bromide (CTAB),^25^ and bovine serum albumin-capped AuNPs on silanization titanium dioxide-modified filter paper (BSA-AuNPs/STCP) in the presence of HBr.^26^ Notably, additional halide-containing chemicals, for example, HBr and CTAB, were mostly used as powerful silver etchants. This halide ion can first interact with Au^0^ or Ag^0^ to generate AuBr_2_^–^ and AgBr, which have a lower standard reduction potential than Cr(VI). Cr(VI) can act as a powerful redox etchant to oxidize Au^0^ and Ag^0^ to Au^3+^ or Ag^+^ species. At the same time, a shape transformation of the nanoparticle occurred during the redox etching reaction, and the correlation between the shape transformation and color development is related to the concentration of Cr(VI). However, there is no report on the use of noble metal NPs in the absence of halide ions to selectively quantify Cr(VI) in water. Therefore, it is challenging to develop metal NP colloids with no addition of halide-containing chemicals for Cr(VI) determination.

In this study, we report a nonhalide etchant system for Cr(VI) detection in tap water using a combination of sodium borohydride-coated gold and citrate-capped silver nanoparticles (Na-AuNPs/cit-AgNPs). During Cr(VI) addition, the redox etching of NPs and particle aggregation induced by Cr(VI) in tap water occurred, resulting in a change in the morphology of both Na-AuNPs and cit-AgNPs. This colorimetric probe exhibited a multicolor transition from the original orange to reddish–purple, dark purplish–red, dark blue–violet, and gray shades. This color transformation directly shows the LSPR shift, as monitored by a UV–vis spectrometer. To the best of our knowledge, there is no report on the utilization of coexisting AuNPs and AgNPs as colorimetric probes for Cr(VI) sensing without the addition of halide ions. Moreover, the demonstration of Cr(VI) determination in a real water sample was also performed.

## 2 Experimental Section

### 2.1 Reagents and Chemicals

Chloroauric acid trihydrate (HAuCl_4_·3H_2_O), trisodium citrate dihydrate (Na_3_C_6_H_5_O·2H_2_O; TSC), sodium borohydride (NaBH_4_), silver nitrate (AgNO_3_), standard heavy-metal solutions (Cr(VI), and Cr(III), Pb(II), Ni(II), Fe(II), Mn(II), As(III), Hg(II), Cd(II), Zn(II), and Co(II)) solutions were purchased from Sigma-Aldrich. All chemicals were purchased from a commercial source and used without further purification. All solutions were prepared using deionized (DI) water (18 MΩ, Smart 2 Pure Ultrapure water system, Thermo Electron LED GmbH, Langenselbold, Germany).

### 2.2 Synthesis of AuNPs

In this study, AuNPs stabilized with two different capping agents (NaBH_4_ and TSC) were separately prepared. The NaBH_4_-capped AuNPs and TSC-capped AuNPs were denoted as Na-AuNPs and cit-AuNPs, respectively. Na-AuNPs were synthesized via a reduction reaction method using NaBH_4_ as a reducing agent.^27^ Briefly, a freshly prepared NaBH_4_ solution in ice-cold water (0.1 M) was rapidly added to 0.25 mM HAuCl_4_ aqueous solution (20 mL). The solution was stirred for 10 min to complete the reduction reaction. Moreover, AuNPs capped with TSC (cit-AuNPs) were synthesized with a protocol similar to the previous method with additional TSC. To do this, 5 mg of TSC was added to HAuCl_4_ aqueous solution, followed by the addition of NaBH_4_ solution. Both Au colloids were left overnight at room temperature before use.

The particle size of Na-AuNPs can be altered by varying the volume of the gold precursor. In this study, four different sizes of AuNPs were prepared, and the volume and concentration of each reagent are given in Table S1.

### 2.3 Synthesis of AgNPs

In this study, AgNPs stabilized with two different capping agents (NaBH_4_ and TSC) were prepared. The NaBH_4_-capped and TSC-capped AgNPs are denoted as Na-AgNPs and cit-AgNPs, respectively. For Na-AgNP synthesis, an as-prepared NaBH_4_ aqueous solution (0.1 M, 0.3 mL) was rapidly injected into 0.3 mM AgNO_3_ aqueous solution (10 mL). To complete the reaction, the mixture was continuously stirred at 700 rpm for 10 min. The solution color changed from brown to light yellow. cit-AgNPs were synthesized by reducing silver salt with TSC, which acts as both the reducing and the capping agent.^28^ Briefly, 0.368 mL of 10 mM AgNO_3_ aqueous solution was mixed with 23.75 mL of DI water. Then, the solution was boiled (100 °C) and stirred at 700 rpm for 10 min. Freshly prepared 1.25 mL of 1% TSC aqueous solution was quickly injected. The color transformed from clear to light yellow after continuous boiling for 30 min, confirming the formation of AgNPs. Colloidal solutions of both Na-AgNPs and cit-AgNPs were stored in the refrigerator until use.

Moreover, the particle size of Na-AgNPs can be tuned by varying the volume of the silver precursor. The volume and concentration of each reagent are given in Table S2.

### 2.4 Determination of Cr(VI)

To determine the concentration of Cr(VI), 75 μL of the AuNP colloid and 25 μL of the AgNP colloid were freshly mixed in 96-well plates; then, 35 μL of the Cr(VI) aqueous solution was added and manually mixed for 10 s. All Au and Ag colloidal solutions were used directly without any washing process. The mixture was incubated at room temperature for 30 min, followed by imaging using the camera of an iPhone and the recording of UV–vis absorption spectra using a UV–vis spectrometer. The sensitivity plot of Cr(VI) detection through the LSPR response was plotted between the absorbance ratio at wavelengths of 600 and 514 nm (*A*_600_/*A*_514_) with respect to the concentration of Cr(VI).

### 2.5 Preparation of Real Samples

Real water samples, including tap water (TW), natural water, and synthetic urine samples, were used without further purification and spiked with the standard Cr(VI) solution at a certain concentration. TW was collected from the laboratory. Natural water was collected from Sriracha, Chonburi Province, Thailand. For synthetic urine samples, urine was diluted with DI water 100 times before being spiked with Cr(VI) standard solution because a high concentration of various kinds of salts dissolved in urine can disturb the Cr(VI) sensing performance.

### 2.6 Instrumentation

The morphology and particle size of metal NPs were characterized by transmission electron microscopy (TEM, Jeol, JEM 2100). An ultraviolet–visible (UV–vis) microplate spectrophotometer (Agilent) was used to observe the optical properties of the samples. The digital image was captured by using an iPhone 13 Pro camera. The electrochemical properties of metal NPs in the detection system were investigated using an electrochemical analyzer-simulator (simulator of chemical sensor, Potentiostat, Zensor, Taiwan).

## 3 Results and Discussion

### 3.1 Characterization of AuNPs and AgNPs

**Figure 1:**
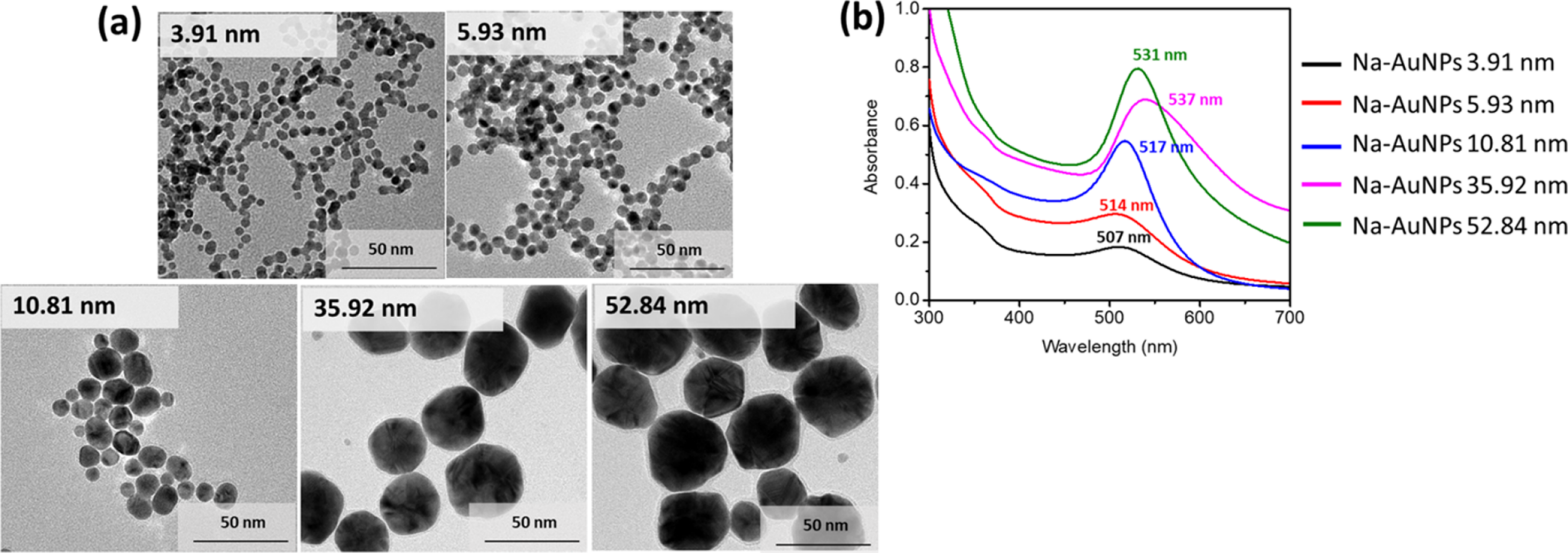
(a) TEM images and (b) extinction spectra of all AuNPs with different particle sizes.

In this study, AuNPs and AgNPs were synthesized through a reduction reaction and separately capped with NaBH_4_ and TSC. The morphology, particle size, and optical properties of both AuNPs and AgNPs were characterized by TEM measurement and UV–vis spectroscopy, respectively. NPs from each synthesis condition were separately reported. Both Na-AuNPs and cit-AuNPs were synthesized through a similar protocol. Therefore, five different particle sizes of AuNPs were varied by changing the amount of gold precursor solution in the synthesis reaction. Figure 1a shows TEM images of all of the Na-AuNPs. All AuNPs were nearly spherical in shape. The size distribution (average diameter ± standard deviation) of all NPs was evaluated from 100 particles in TEM images using ImageJ software. The particle sizes of both Na-AuNPs and cit-AuNPs were similar when compared to the same synthesis protocol. Therefore, the addition of a TSC capping agent in the reaction has no effect on the particle size. The diameters of AuNPs were estimated to be about 3.91 ± 0.53, 5.93 ± 0.64, 10.81 ± 5.89, 35.92 ± 6.19, and 52.84 ± 13.12 nm when the volumes of the HAuCl_4_ aqueous solution were increased to 0.25, 0.5, 0.6, 0.75, and 1 mL, respectively. The particle size increased as the amount of gold precursor was increased. Figure 1b shows the extinction spectra of all of the Na-AuNPs. The maxima of the extinction band assigned as the localized surface plasmon resonance (LSPR) of all Na-AuNPs were about 507, 514, 517, 531, and 537 nm for Na-AuNPs at particle sizes of 3.91, 5.93, 10.81, 35.92, and 52.84 nm, respectively. The LSPR increased with increasing particle size. The LSPR peak red shift was induced by an increase in the AuNP diameter.^29^ The original color of AuNPs at each diameter was pale pinkish–orange, pinkish–orange, red, cherry–red, and purplish–red for 3.91, 5.93, 10.81, 35.92, and 52.84 nm, respectively. The images of all AuNPs are shown in Table S1.

**Figure 2:**
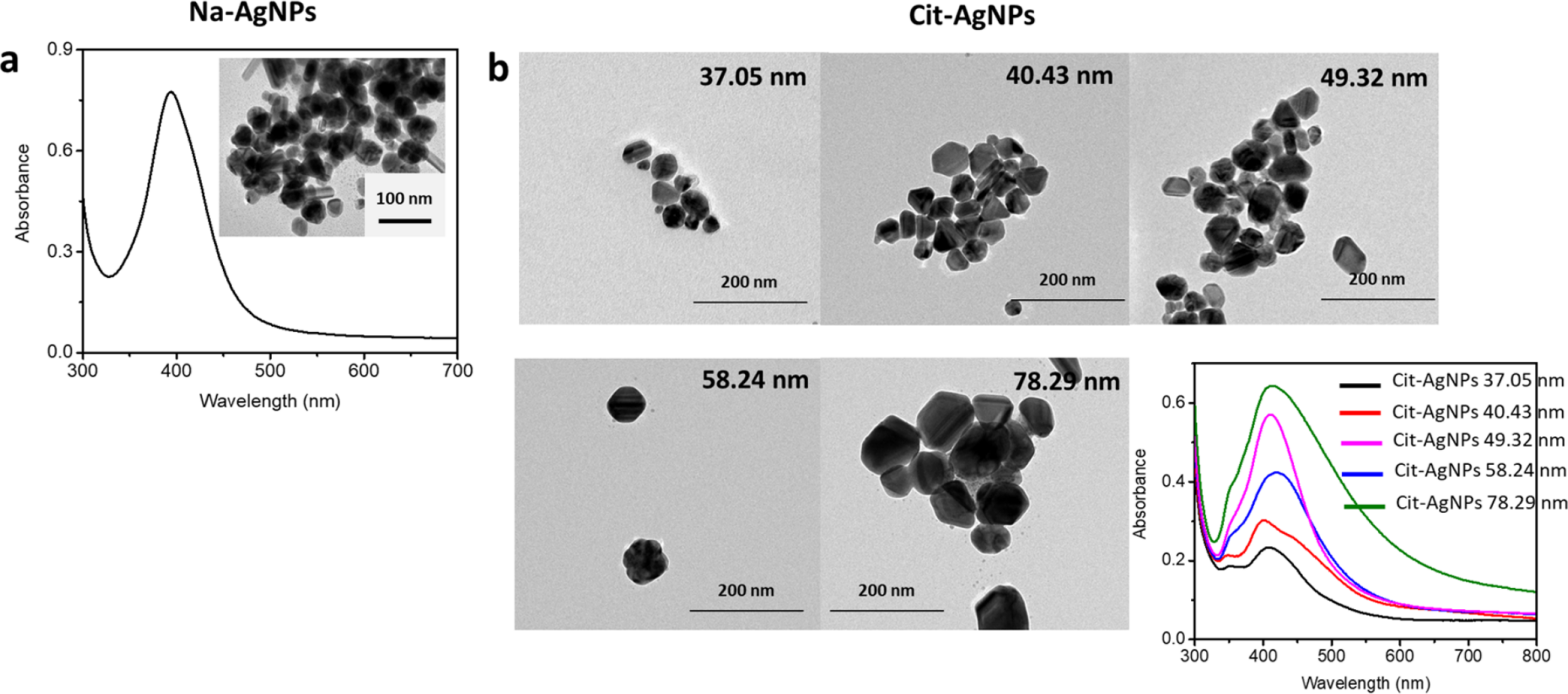
TEM images and extinction spectra of (a) Na-AgNPs and (b) all cit-AgNPs with different particle sizes.

In the case of AgNP synthesis, both Na-AgNPs and cit-AgNPs were prepared by different protocols. Therefore, Na-AgNPs and cit-AgNPs were separately explained. Figure 2a shows the UV–vis spectrum, and the inset displays the TEM image of Na-AgNPs. For Na-AgNPs, the particle size was about 44.53 ± 6.28 nm and LSPR was located at about 398 nm. In the case of cit-AgNPs, five different particle sizes were tested, and the estimated particle sizes were about 37.05 ± 6.45, 40.43 ± 6.83, 49.32 ± 8.55, 58.24 ± 10.47, and 78.29 ± 16.87 nm with an increase in the silver solution to 0.276, 0.368, 0.552, 0.736, and 1.472 mL, respectively (Figure 2b). The extinction spectra of cit-AgNPs with different particle sizes exhibit a similar LSPR at about 400–411 nm. The bandwidth increased as the particle size of cit-AgNPs increased.^30^ Both Na-AgNPs and cit-AgNPs exhibited similar trends with the same LSPR position. The original color of AgNPs of all diameters was yellow, as mentioned in Table S2.

### 3.2 Feasibility Test of Each AuNP and AgNP for Cr(VI) Sensing in TW

It is very challenging to prepare stable AuNPs or AgNPs to detect Cr(VI) contamination in real TW. The presence of various ions in TW is the reason for the formation of aggregates in both AuNPs and AgNPs. This is a crucial obstacle to the use of AuNP and AgNP colloids as colorimetric probes. In this study, 5.93 nm AuNPs and 40 nm AgNPs with two different capping agents of NaBH_4_ and TSC were selected as probes. Each colloidal system (100 μL) was tested separately by adding DI and TW (50 μL). The characteristic behavior of each probe was investigated using its UV–vis spectra and images. Figure S1 shows the extinction spectra of each AuNP and AgNP in the presence of DI and TW. The digital image (inset on the right of Figure S1) displays the color transformation of each colloid after being mixed with DI and TW. Upon mixing with TW, both Na-AuNPs and Na-AgNPs exhibit a red shift in the LSPR along with a change in color. For the Na-AuNPs system, the LSPR characteristic band at 517 nm was red-shifted to two absorption bands at 521 and 612 nm with a broad absorption. This shift corresponds to the color transformation from the original orange-red color of Na-AuNPs to purple when mixed with TW. In the case of Na-AgNPs, the single LSPR peak at 398 nm obviously changed into two absorption bands at 384 and 531 nm. These solution colors changed from yellow to orange–red. This phenomenon may possibly be caused by the aggregation of both AuNPs and AgNPs capped with NaBH_4_ in the presence of complex components of TW. However, both cit-AuNPs and cit-AgNPs show good stability, as no changes were observed in both the LSPR band and the solution color. This is explained by the fact that the capping agent plays an important role in the stability of AuNPs and AgNPs in the presence of TW.

### 3.3 Effect of the AuNP/AgNP Mixture on Cr(VI) Detection

**Figure 3:**
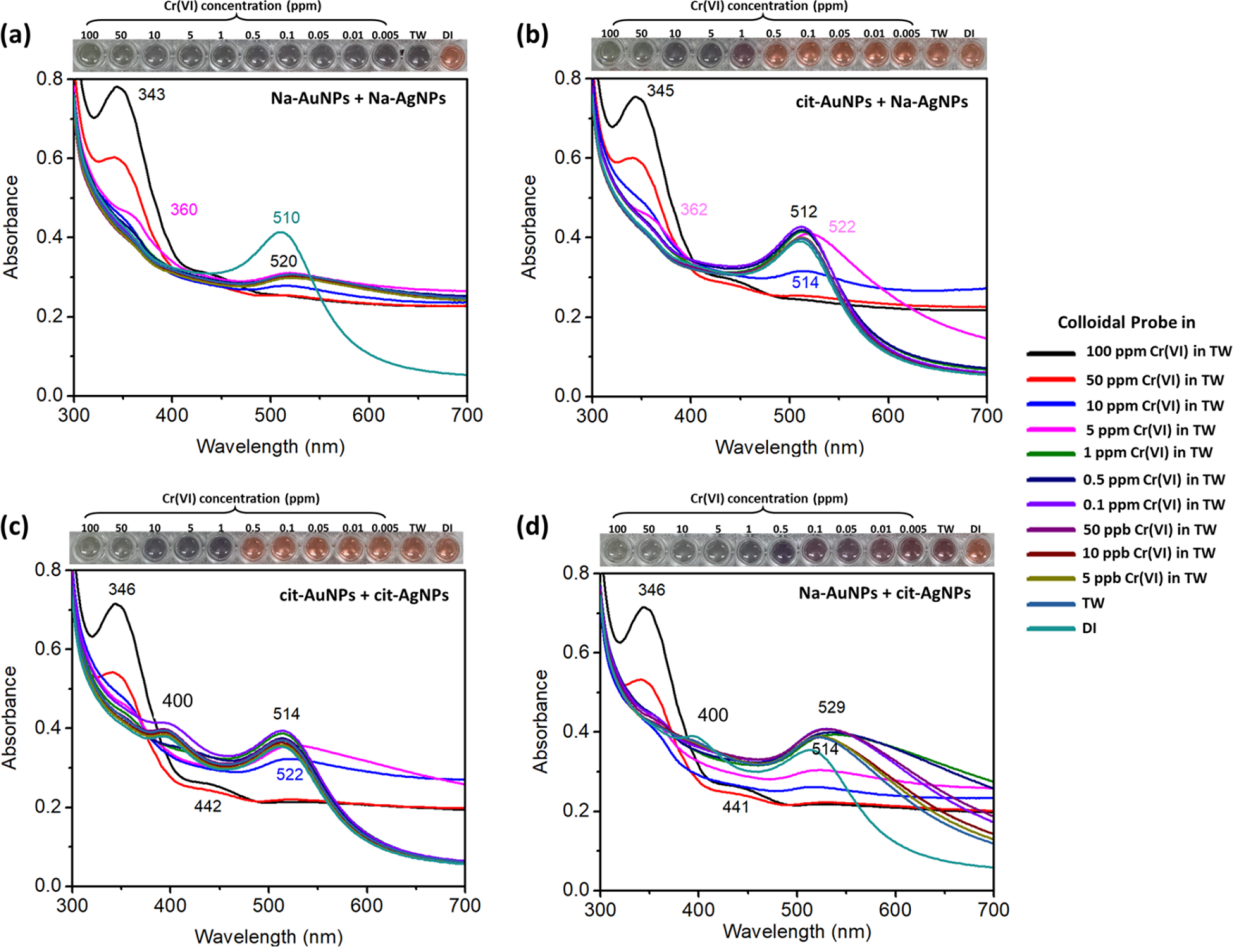
Extinction spectra, with the inset showing the corresponding digital images of the probe mixture in the presence of Cr(VI) in TW: (a) Na-AuNPs/Na-AgNPs, (b) cit-AuNPs/Na-AgNPs, (c) cit-AuNPs/cit-AgNPs, and (d) Na-AuNPs/cit-AgNPs.

In a previous experiment, the stability of the single-metal NP system, especially the NaBH_4_ system, was not stable after being mixed with TW. In this study, the mixed solution of AuNPs and AgNPs was used as a colorimetric probe for its capability of sensing TW contaminated with Cr(VI). Four different colloidal mixtures were prepared, including (i) Na-AuNPs/Na-AgNPs, (ii) cit-AuNPs/Na-AgNPs, (iii) cit-AuNPs/cit-AgNPs, and (iv) Na-AuNPs/cit-AgNPs. The particle sizes of AuNPs and AgNPs were fixed at 5.93 and 40 nm, respectively, for observing only the effect of the AuNPs/AgNPs combination. The volume ratio between AuNPs and AgNPs was set to be 3:1 to obtain a final volume of 100 μL. The Cr(VI) in TW (50 μL) was added to the previous colloid and mixed for 30 min. The concentration of Cr(VI) was varied from 5 ppb to 100 ppm. TW was used as received without any treatment or dilution. The detection characteristics were investigated by observing the color transformation and UV–vis spectra of the AuNPs/AgNPs probe combination after reacting with different Cr(VI) concentrations. Figure 3 depicts the extinction spectra, with the inset showing the image of probe mixtures in the presence of Cr(VI) in TW. The mixture of AuNPs and AgNPs coated with different capping agents shows different detection behaviors, as seen from both UV–vis spectra and digital images. The original colors of all AuNPs/AgNPs probe combinations were similar, as indicated by the orange color in the right column of all digital images. For the mixed colloid of Na-AuNPs and Na-AgNPs (Figure 3a), the original orange color changed to gray color after mixing with only TW and gradually transformed to a pale gray and finally pale green color as the Cr(VI) content increased. In addition, the LSPR characteristic peaks of both AuNPs (514 nm) and AgNPs (398 nm) drastically reduced after mixing with Cr(VI). An additional LSPR band at a lower wavelength of about 345 nm was observed. The cutoff level for Cr(VI) detection was hardly distinguishable by the naked eye. Therefore, this mixture solution is not suitable for further use as a probe for Cr(VI) sensing in TW. In the case of cit-AuNPs/cit-AgNPs (Figure 3b) and cit-AuNPs/Na-AgNPs mixtures (Figure 3c), a high Cr(VI) content (1–100 ppm) can remarkably alter both the color and LSPR properties. The solution color transformed from the original orange color to purple and pale gray. UV–vis spectra of these two detection systems were comparable, as indicated by a single additional LSPR peak at about 345 nm (Figure 3b,3c). No difference in color transformation was observed by the naked eye, and less reduction of absorbance was found when probe colloids reacted with a low Cr(VI) content (0–0.5 ppm). These two AuNPs/AgNP combinations can be used as colorimetric probes for high Cr(VI) content sensing with a cutoff level of 5 ppm detected by the naked eye. Surprisingly, the mixture solution of Na-AuNPs and cit-AgNPs (Figure 3d) showed a multicolor variation after reaction with Cr(VI) at different concentrations. The probe color changed from the original orange to a pale reddish-purple when mixed with TW at a low Cr(VI) content (5–10 ppb). The solution developed a dark reddish-purple color when mixed with Cr(VI) at 50–100 ppb. A dark bluish-purple color was observed when the probe solution reacted with 0.5 and 1 ppm of Cr(VI). The probe changed to dark gray and finally to a pale greenish-gray color when mixed with a high Cr(VI) content (5–100 ppm). The spectral response of the LSPR of the mixed Na-AuNPs/cit-AgNPs combination (Figure 3d) was sensitive to the presence of Cr(VI) over the entire measurement range. In the beginning, Na-AuNPs/cit-AgNPs showed two absorption bands at 400 and 514 nm, corresponding to the characteristic LSPR peak of cit-AgNPs and Na-AuNPs, respectively. After mixing TW with the probe solution, the absorbance of both metal NPs slightly decreased with a wide absorption wavelength. Weak LSPR peaks with a wider absorption wavelength were observed as the Cr(VI) concentration increased. No characteristic LSPR bands of both AuNPs and AgNPs were found for the reaction of the probe at a very high concentration of Cr(VI) (50 and 100 ppm). Additional absorbance at a lower wavelength of 346 nm was observed. A linear response can possibly be created from the relationship between the ratio of the absorbance at 600:514 (*A*_600_/*A*_514_) with respect to the Cr(VI) content. According to these finding results, this Na-AuNPs/cit-AgNPs combination shows a novel system for using the mixture of AuNPs and AgNPs as a colorimetric probe for Cr(VI) detection. This probe system can directly react with Cr(VI) without any additional powerful etchants, including halides or acid-containing molecules. This is an advantage over other previous reports on the utilization of noble metal NP coatings with various types of stabilizing agents in the presence of halide ions.^26,31−34^ Therefore, the suitable detection system of the Na-AuNPs/cit-AgNP combination was selected for further study.

### 3.4 Optimization of Na-AuNPs/cit-AgNPs Combination for Cr(VI) Ion Detection in TW

To achieve a suitable probe colloid for the highly sensitive detection of Cr(VI) ion-contaminated TW, the optimization of Na-AuNPs/cit-AgNP combination, including the particle sizes of Na-AuNPs and cit-AgNPs, the optical density of Na-AuNPs, the ratio of Au/Ag, and the volume of Cr(VI) ion in tap water, were studied. As the detailed investigation of a suitable Na-AuNPs/cit-AgNP combination for Cr detection is described in the SI, here, only brief results are presented.

First, different particle sizes of Na-AuNPs (3.91, 5.93, 10.81, 35.92, and 52.84 nm) were synthesized by altering the amount of gold precursor in the synthesis reaction. The particle size of cit-AgNPs was fixed at about 40 nm. The volume ratio of AuNPs/AgNPs was fixed at 3:1 (final volume = 100 μL) and the volume of Cr(VI) was fixed at 50 μL. The concentration of Cr(VI) ranged from 0.1 to 100 ppm. UV–vis spectra and images depicting the AuNPs/AgNPs combination upon the addition of Cr(VI) are displayed in Figure S3. All AuNPs/AgNPs combinations with various sizes of Na-AuNPs show no aggregation upon the addition of Cr(VI) ions. The efficacy of Cr(VI) detection varied with the particle sizes of Na-AuNPs. The probe combination of Na-AuNPs (5.93 nm)/cit-AgNPs exhibited a highly sensitive response toward Cr(VI) determination as observed by the multicolor transformation from orange to purple, blue, and finally green color. This system showed the best response toward Cr(VI) detection. Consequently, Na-AuNPs (5.93 nm) were selected for further investigation.

Subsequently, the particle size of cit-AgNPs was also varied (37.05, 40.43, 49.32, 58.24, and 78.29 nm) by changing the silver precursor in the synthesis reaction. 5.93 nm Na-AuNPs were chosen to mix with cit-AgNPs to form a probe colloid. The AuNPs/AgNPs/Cr volume ratio was kept at 3:1:2 (with a final volume of 150 μL). The behavior of the probe during Cr(VI) detection was monitored by collecting UV–vis spectra and images of the solution. Figure S4 depicts the UV–vis spectra and digital images of the Au/Ag probe solution using various particle sizes of cit-AgNPs upon Cr(VI) addition. In the digital images, multicolor evolution from the original orange to old-rose, dark purple, dark blue, and finally gray color was observed for all sizes of cit-AgNPs with different detection ranges. It was determined that a broader detection range (0.5–100 ppm) was achieved when using cit-AgNPs with sizes of 37.05, 40.43, and 49.32 nm (Figure S4a–c). However, larger cit-AgNPs (58.24 and 78.29 nm) exhibited a narrow detection range at Cr(VI) concentrations of 0.5–10 ppm (Figure S4d,e). As a result, a mean diameter of 40.43 nm was chosen as the optimal size of cit-AgNPs for subsequent investigations.

Upon achieving the appropriate sizes of both Na-AuNPs (5.93 nm) and cit-AgNPs (40.43 nm), these particles demonstrated a wide detection range for Cr(VI) detection. However, it was difficult to distinguish the color shade by the naked eye because of the light-colored probe colloid. Enhancing the detection limit for naked-eye observation is crucial. It is assumed that the color evolution of this probe colloid mainly comes from the morphological change of AuNPs, which directly affects the alteration of the absorption behavior of particles. Therefore, an intense probe color was developed by increasing the concentration of Na-AuNPs. The concentration of Na-AuNPs was adjusted by increasing both the gold precursor and the reducing agent in the synthesis reaction. Detailed information regarding the synthesis protocol and characterization of the AuNP concentration variation is provided in Table S3 and Figure S2. The concentration of as-synthesized Na-AuNPs was monitored from the optical density (OD) of the Au colloid obtained from UV–vis spectra. The OD value varied from 0.30 to 1.76. The Cr(VI) monitoring system was prepared by fixing the volume ratio of AuNPs/AgNPs/Cr at 3:1:2 (final volume 150 μL). Figure S5 represents the UV–vis spectra and digital images of the Au/Ag probe solution at different concentrations of Na-AuNPs upon Cr(VI) addition. The color intensity of the probe colloid increased as the concentration of Na-AuNPs increased. For the LSPR response of the probe colloid upon Cr(VI) addition, the variation in the absorption band of Na-AuNPs was observed with varying concentrations of Na-AuNPs. By achieving a balance between visual observation and the LSPR response, the probe colloid containing Na-AuNPs at an OD of 0.87 was selected as an optimum Na-AuNP concentration for further investigation. This is because the response of color transformation upon Cr(VI) addition was easily distinguished by the naked eye, and the linear response can possibly be created from the plot between the absorbance ratio at 600:517 (*A*_600_/*A*_517_) and the Cr(VI) content.

Moreover, the quantity of analyte is a critical factor influencing the color transformation and absorption spectra of the probe upon the addition of Cr(VI). In this investigation, the volume of Cr(VI) solution was varied from 25 to 35, 50, and 75 μL and added to the probe colloid (with a fixed AuNPs/AgNPs volume ratio of 3:1; total 100 μL). Both the color change and the optical properties were monitored upon the addition of Cr(VI) in the concentration range of 0.01–100 ppm. Figure S6 shows the UV–vis spectra and digital images of the Au/Ag probe solution at different volumes of Cr(VI) addition. By considering the digital image, the detection system with different Cr(VI) volumes exhibited different color evolutions of the probe colloid. A small amount of the analyte (25 μL) (Figure S6a) may not be sufficient to elicit a wide detectable color difference by the naked eye. For a large volume of Cr(VI) solution (50 and 75 μL) (Figure S6c,d), the color of the probe colloid changed to dark purple and gray when mixed with Cr(VI), and it was difficult to determine the detection limit. When 35 μL of Cr(VI) solution was added to the probe colloid (Figure S6b), multicolor transformation from orange to dark red, purple, blue, and finally gray–green color was found for Cr(VI) detection in the range of 0.05–100 ppm. Additionally, the absorption peak at longer wavelengths (>600 nm) increased with increasing Cr(VI) volume. This is caused by the aggregation of AuNPs induced by the high content of Cr(VI). This result is in good agreement with the result of color development from the digital images. According to the result, a Cr(VI) volume of 35 μL provided a favorable system for the visual detection process, which was chosen as an optimum Cr(VI) content for further investigation.

Finally, determining the appropriate component ratio of the probe colloid, comprising Na-AuNPs and cit-AgNPs, emerges as a crucial parameter for achieving highly sensitive detection. The volume ratio between AuNPs and AgNPs varied from 9:1 to 3:1 and 1:1 (resulting in a final volume of 100 μL). The standard Cr(VI) spiked in TW in the concentration range of 0.01–100 ppm was used as the analyte. A fixed volume of Cr(VI) solution (35 μL) was added to the previous probe colloid. The detection performance was investigated by observing the color evolution and UV–vis spectra of the probe at different AuNPs/AgNPs ratios after reacting with different Cr(VI) concentrations. Figure S7 illustrates the extinction spectra, with the inset showing the corresponding digital image of the probe colloid at three different Au/Ag volume ratios upon the addition of Cr(VI). Each colorimetric probe system exhibited different spectral changes and color transformations. It was found that an unidentified detection range was obtained from the probe colloid containing a high Na-AuNPs content (Au/Ag ratio = 9:1) (Figure S7a), indicated by a dark purple and gray color when mixed with Cr(VI). The sensitivity of this probe colloid could not be enhanced by increasing the amount of Na-AuNPs. A probe colloid with an Au/Ag volume ratio of 1:1 (Figure S7c) exhibited a narrow visual detection range at a high Cr(VI) content (5–100 ppm), indicated by the color development from orange to dark purple and gray when reacted with Cr(VI). The absorption band of both cit-AgNPs and Na-AuNPs gradually decreased with increasing Cr(VI) content (0–1 ppm). For the probe colloid prepared by mixing Na-AuNPs and cit-AgNPs at a 3:1 volume ratio (Figure S7b), the multicolor evolution (original orange to reddish–purple, dark purplish–red, dark blue–violet, and gray color) with a wide visual detection range from 0 to 100 ppm of Cr(VI) concentration was observed. The LSPR peak of Na-AuNPs gradually decreased with wide absorption wavelength as the Cr(VI) concentration increased (from 0 to 10 ppm). Subsequently, the characteristic LSPR bands of both AuNPs and AgNPs disappeared, and the addition absorption band at about 345 nm was found when reacted with a high Cr(VI) concentration at 50–100 ppm. This probe system exhibited a correlation between the spectral changes in the AuNP bands upon the addition of Cr(VI), potentially enabling the quantitative determination of Cr(VI). Furthermore, it achieved high sensitivity for Cr(VI) detection by visual observation. Considering all optimization results, the suitable detection system comprises (1) a probe colloid consisting of a mixture of 5.93 nm Na-AuNPs and 40.43 nm cit-AgNPs at a fixed volume ratio of AuNPs/AgNPs of 3:1 and (2) an optimum Cr(VI) volume of 35 μL. These parameters were employed for further analytical performance studies.

### 3.5 Mechanism of this Sensor toward Cr(VI) Detection

**Figure 4:**
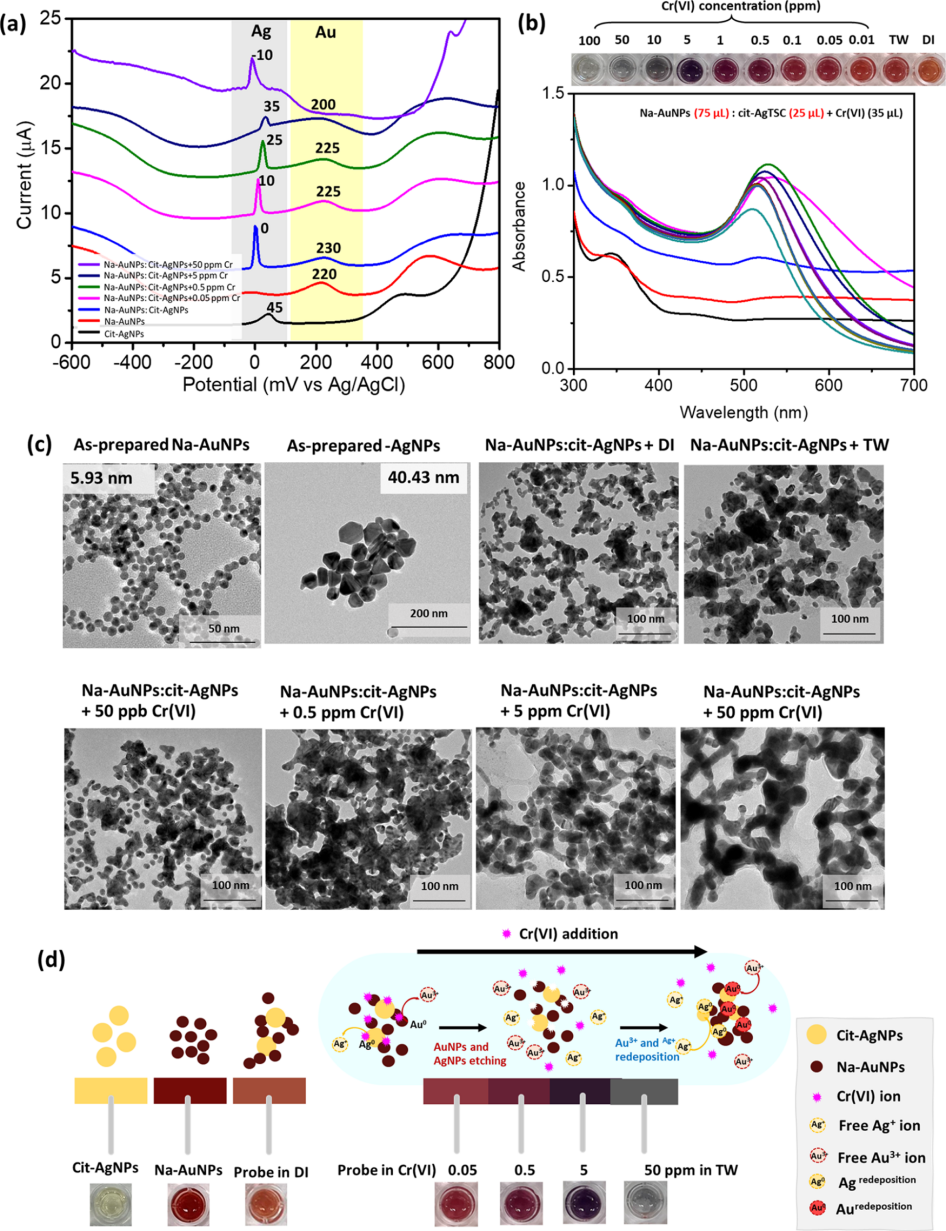
(a) Differential pulse voltammograms on bare screen-printed carbon electrodes. (b) Extinction spectra and digital image. (c) TEM images of the Na-AuNPs/cit-AgNPs probe colloid in the absence and presence of Cr(VI) in the concentration range of 0–50 ppm. (d) The plausible mechanism of Cr(VI) ion detection using the Na-AuNPs/cit-AgNPs probe colloid. The color change of probe colloids after reacting with different concentrations of Cr(VI) dissolved in TW.

To understand the Cr(VI) sensing mechanism of this probe mixture, the probe solution in the presence of different Cr(VI) contents was characterized by performing electrochemical, UV–vis, and TEM measurements. The electrochemical method is a useful technique to understand the nature of each metal particle and confirm our sensing mechanism. In this study, an electrochemical differential pulse voltammetry (DPV) measurement was conducted on the probe solution in both the absence and the presence of Cr(VI) ions in TW. The detection procedure using the electrochemical method is detailed in SI. Figure 4a shows differential pulse voltammograms of pristine Na-AuNPs and cit-AgNPs and their mixture in the presence of Cr(VI) ion in TW. The native cit-AgNPs and Na-AuNPs exhibit single oxidation peaks at potentials of 45 and 220 mV (vs Ag/AgCl), respectively. The mixed Na-AuNPs and cit-AgNPs colloid shows two oxidation peaks at potentials of 0 and 230 mV (vs Ag/AgCl), which correspond to the oxidation peaks of cit-AgNPs (Ag^0^) and Na-AuNPs (Au^0^), respectively. The reduction in the oxidation potential of cit-AgNPs is attributed to the particle size reduction resulting from galvanic replacement between residual Au^3+^ in the Na-AuNPs colloid and Ag^0^ of cit-AgNPs. The reaction produces Ag^+^ ions as byproducts, which can possibly be deposited on the SPCE surface or AuNPs, forming small AgNPs. Simultaneously, an increase in the oxidation potential of AuNPs resulted from the enlargement of Na-AuNPs or their aggregates. Upon the addition of a Cr(VI) ion solution, both the oxidation peak potential and the current signal of Na-AuNPs and cit-AgNPs undergo significant changes. At low concentrations of Cr(VI) (0.05–5 ppm), the oxidation peak of cit-AgNPs shifted to a higher potential from 0 to 35 mV and with a somewhat lower current intensity than the pristine probe solution (Na-AuNPs/cit-AgNPs). In addition, the oxidation peak of AuNPs gradually shifted to a lower potential (from 230 to 200 mV), with a lower current signal when reacted with a low Cr(VI) concentration (0.05–5 ppm). This can be explained by the initial etching of AuNPs (Au^0^) to smaller AuNPs and Au^3+^ ions, induced by the higher standard redox potential of Cr^6+^ compared to that of Au^3+^ (Cr^6+^ /Cr^3+^ = 1.36 V, Au^3+^/Au^0^ = 1.002 V).^35,36^ Then, free Au^3+^ ions can be deposited on cit-AgNPs and possibly etch AgNPs to form larger AgAuNPs, induced by the higher standard redox potential of Au^3+^ than that of Ag^+^ (Ag^+^/Ag^0^ = 0.7996 V, Au^3+^/Au^0^ = 1.002 V).^37^ At a high Cr(VI) concentration (50 ppm), an excess amount of Cr(VI) can significantly etch both Na-AuNPs and cit-AgNPs, as evidenced by the observation of the oxidation peak of cit-AgNPs at a low potential of approximately −10 V with a higher current signal and the absence of an oxidation peak of Na-AuNPs. The high content of Cr(VI) can effectively dissolve Na-AuNPs and cit-AgNPs into Au^3+^ and Ag^+^ in the detection system.

Furthermore, the optical properties of the probe colloid (Na-AuNPs/cit-AgNPs) were monitored as a function of Cr(VI) concentration in TW using UV–vis measurement and digital images. Figure 4b shows the extinction spectra of the probe colloid (Na-AuNPs/cit-AgNPs) in the absence and presence of the Cr(VI) ion, along with their corresponding digital images. Analysis of the UV–vis spectra reveals that Cr(VI) can directly interact with both Na-AuNPs and cit-AgNPs through different mechanisms. Upon Cr(VI) addition (0.01–100 ppm), the extinction peak of cit-AgNPs at 400 nm visibly diminishes, indicating that Cr(VI) can catalyze the oxidation reaction of Ag^0^ to form Ag_(aq)_^+^ in the solution.^21^ Simultaneously, an excess amount of Cr(VI) can induce AuNP aggregation as indicated by a reduction of the AuNP absorption band (λ = 514 nm), accompanied by a broad absorption peak (>550 nm) and the formation of black precipitates of AuNPs in the solution.^20^ The broad absorption band observed in AuNPs indicates the presence of both individual nanoparticles and aggregates in the solution. With the addition of high concentrations of Cr(VI) (50–100 ppm), the characteristic LSPR bands of both Na-AuNPs and cit-AgNPs at about 514 and 400 nm, respectively, clearly disappeared. This confirms the dissolution of both AuNPs and AgNPs by Cr(VI). This observation aligns well with the results obtained from the electrochemical measurements. In addition, the probe color changed from its original orange to reddish-orange when mixed with TW at a low Cr(VI) content (0–10 ppb). The solution developed a reddish-purple color when mixed with Cr(VI) at 50–100 ppb. A dark purplish-red color was observed for the probe solution when reacted with 0.5–1 ppm of Cr(VI). As the Cr(VI) content increased, the color of the probe transformed to a dark blue-violet and eventually to a pale gray color with black precipitation when mixed with higher Cr(VI) concentrations at 5 and 10–100 ppm, respectively. These findings demonstrate the multicolor transformation of the probe colloid upon the addition of Cr(VI) to contaminated TW.

To derive more information about the mechanism of the probe colloid toward Cr(VI) detection, the morphology of the probe upon the addition of Cr(VI) at different concentrations was observed using TEM measurements. Figure 4c exhibits the TEM images of the Na-AuNPs/cit-AgNPs probe colloid in the absence and presence of Cr(VI). Furthermore, the relationship between the alteration in the particle size of both AuNPs and AgNPs was monitored upon the addition of Cr(VI) at varying concentrations. Figure S8 reveals the double *y*-axes plot, showing the diameter of Na-AuNPs (on the left) and the diameter of cit-AgNPs (on the right) versus the probe colloid reacted with different concentrations of Cr(VI) in TW. Initially, the morphology of freshly mixed Na-AuNPs and cit-AgNPs in deionized water (DI) was observed to understand the behavior of this probe colloid without the addition of Cr(VI) and TW. The small particles of Na-AuNPs are preferably located near the larger cit-AgNPs surface. An increase in the AuNP particle size was observed, while the diameter of AgNPs slightly decreased (Figure S8). These findings are consistent with the results obtained from electrochemical measurements. Upon considering the probe colloid in the presence of TW, both AuNPs and AgNPs appear to be larger compared to those in the deionized water (DI) system. This may be attributed to the presence of numerous ions in TW, which could induce the aggregation of both particles. After mixing the probe colloid with a low content of Cr(VI) in TW (50 ppb), the diameter of AgNPs slightly decreased. Besides, the population of both single AuNP particles and small aggregates increased.

It is explained that a small amount of Cr(VI) can directly react with both AgNPs and AuNPs, resulting in a partial dissolution of both particles by the redox etching mechanism. This increase of individual and small aggregates of AuNPs possibly alters the shade of the probe solution from the original orange to a reddish-purple color (see inset of Figure 4b). By considering the probe colloid reacted with 0.5 ppm of Cr(VI)-contaminated TW, it was found that both AuNPs and AgNPs were connected to each other, resulting in the aggregation and the dark purplish–red color of the probe (see inset of Figure 4b). By increasing the Cr(VI) content (5 ppm) in the probe colloid, the particle size of AgNPs significantly decreased, as indicated by the small size of dark particles in the TEM image. Moreover, individual AuNPs merged to form rod-like or tube-like particles. This morphological transformation influences the optical properties of the probe colloid, as indicated by a dark blue–violet color (see inset of Figure 4b).

The observation of the probe colloid reacted with TW contaminated with a very high concentration of Cr(VI) (50 ppm) showed that the morphologies of both native AuNPs and AgNPs completely transformed into web-like nanostructures. The diameter of the web was approximately 25–50 nm. This formation of a net structure induced blank precipitation, as observed in the inset of Figure 4b, and the probe solution turned gray. According to these results from electrochemical, optical, and morphological measurements, all data showed a good correlation. Based on these experimental findings, a plausible mechanism for Cr(VI) ion detection was proposed. It is assumed that the redox etching mechanism between Cr(VI) and AgNPs or AuNPs serves as the driving force for this sensor. The redeposition of free Ag^+^ or Au^3+^ ions on the AgNPs or AuNPs and their aggregations may consequently occur. These approaches strongly influence the morphology and color transformation of the probe colloid. Figure 4d exhibits the proposed mechanism for Cr(VI) determination using the Na-AuNPs/cit-AgNPs probe colloid.

### 3.6 Analytical Performance of the Colorimetric Probe System Developed by us toward Cr(VI) Detection in TW

**Figure 5:**
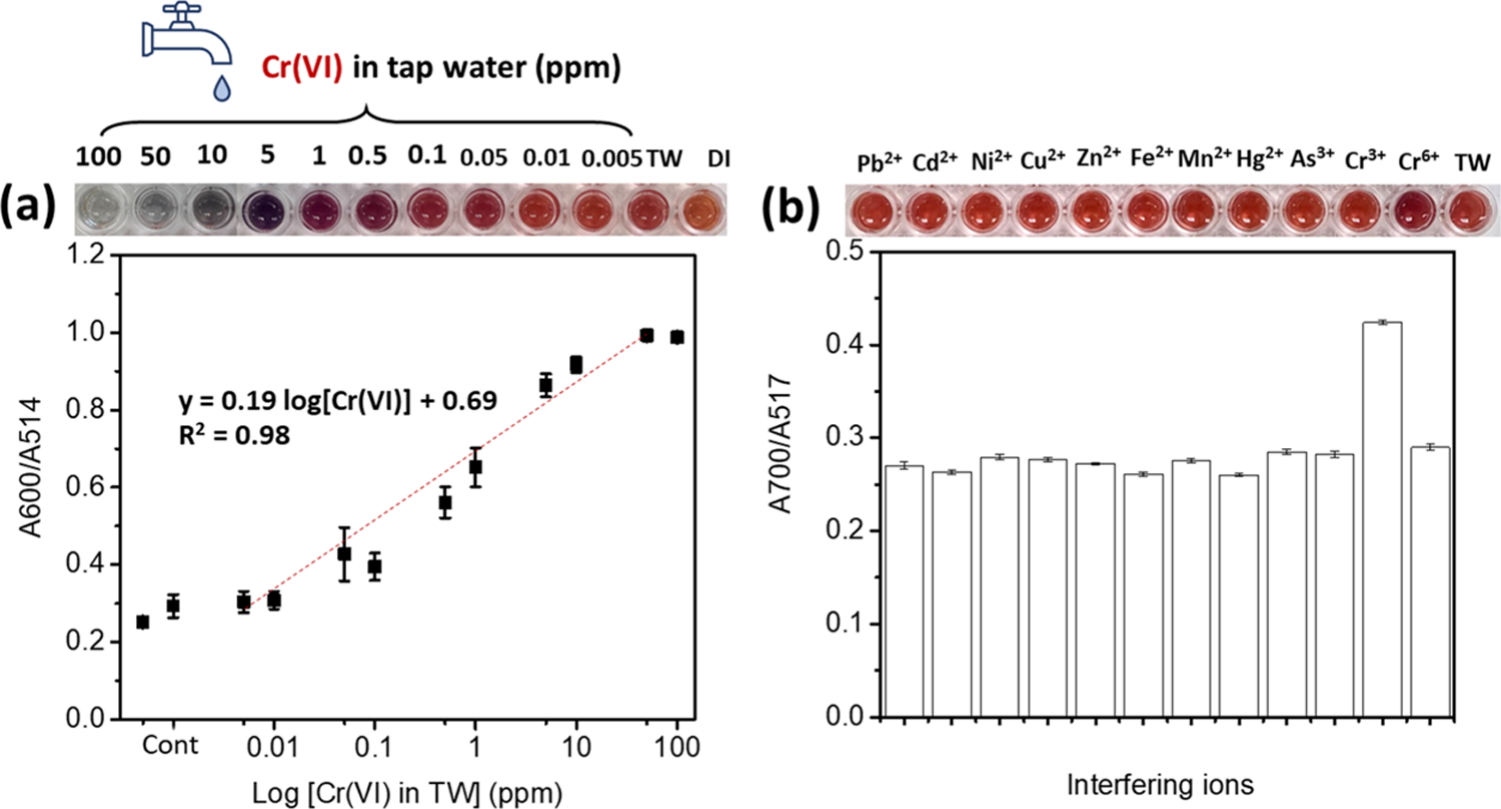
(a) Sensitivity plot of the absorbance ratio at wavelengths of 600 and 517 nm (*A*_600 nm_/*A*_517 nm_) with respect to the concentration of Cr(VI). The dashed straight lines are obtained by the least-squares method. (b) Selectivity of our Na-AuNPs/cit-AgNPs probe colloid. The concentration of all interfering ions was fixed at 0.1 ppm. The absorbance ratio at wavelengths of 600 and 517 nm was plotted against HM ions.

The analytical performance of a colorimetric probe system, including sensitivity, selectivity, and stability, was studied under the optimum conditions through both UV–vis measurement and naked eye detection. A mixture of 5.93 nm Na-AuNPs and 40.43 nm cit-AgNPs was selected for the demonstration of Cr(VI) sensing in TW. To quantitatively determine Cr(VI) sensing, 10 different concentrations of Cr(VI) were serially prepared in TW, and the absorption spectra and color changes were monitored. The linear response was plotted between the absorption ratio (*A*_600_/*A*_517_) and the logarithm of Cr(VI) concentration (Figure 5a). Images of the probe colloid at each Cr(VI) concentration are shown in the inset of Figure 5a. The corresponding spectral response of the probe for the entire measurement range of Cr(VI) detection is provided in FigureS9. The linear response was found in the Cr(VI) concentration ranging from 5 ppb to 10 ppm. The linear relationship is displayed by the linear equation *y* = 0.19 log[Cr] + 0.69, with a good correlation coefficient of *R*^2^ = 0.98. The limit of detection (LOD) of Cr(VI) was estimated based on our previous report.^38^ The LOD was approximately about 22.9 ppb, which was lower than the acceptable Cr level in drinking water issued by EPA (50 ppb).^39^ In addition, the metallic NP-based colorimetric sensor toward Cr(VI) sensing developed by us and reported here yielded a low LOD of 50 ppb from visual observation. Table S3 summarizes a comparison of other previous reports using different types and morphologies of metallic NPs toward Cr(VI) detection. The simple fabrication, low cost, good sensitivity, and no addition of other chemicals are the merits of the metallic NPs-based colorimetric sensor developed by us over other previous reports, which require complicated synthesis steps and skilled operation.

The selectivity of this developed colorimetric probe colloid was studied. Many common metal ions, including Pb^2+^, Cd^2+^, Ni^2+^, Cu^2+^, Zn^2+^, Fe^2+^, Mn^2+^, Hg^2+^, As^3+^, and Cr^3+^, were examined. The experiment was performed at a fixed concentration of all metal ions at 0.1 ppm, and all ions were dissolved in TW. The colloidal probe mixed with TW was also prepared as a control. Figure 5b and the inset show the absorbance ratio (*A*_600_/*A*_517_) and image, respectively, of the probe colloid in the presence of other HM ions. The corresponding extinction spectra of the selectivity study are provided in Figure S10. It was found that a wide absorption band and reddish–purple color were only observed when the probe colloid reacted with Cr^6+^ ions, while Pb^2+^, Cd^2+^, Ni^2+^, Cu^2+^, Zn^2+^, Fe^2+^, Mn^2+^, Hg^2+^, As^3+^, and Cr^3+^ showed results comparable to the control sample (TW), as indicated by an insignificant change in both the UV–vis spectra and the color of the probe colloid. This result indicates the good selectivity of the probe colloid toward Cr(VI) detection.

The stability of Na-AuNPs and cit-AgNPs was examined by monitoring the UV–vis spectra over 4 months of storage. Both colloids were stored in a refrigerator and sampled monthly for UV–vis analysis. Figure S11a,b depicts the UV–vis spectra with respect to the storage time of Na-AuNPs and cit-AgNPs, respectively. Single absorption bands at 517 and 402 nm, without additional peaks at higher wavelengths, were observed for Na-AuNPs and cit-AgNPs, respectively. The shape of the extinction spectra of both Na-AuNPs and cit-AgNPs remained the same as that of the freshly prepared colloids. This suggests that the colloidal solutions of both Na-AuNPs and cit-AgNPs remained stable throughout the 4 month storage period.

### 3.7 Feasibility Test of Our Colorimetric Probe System for the Analysis of Other Real Sample Media

**Figure 6:**
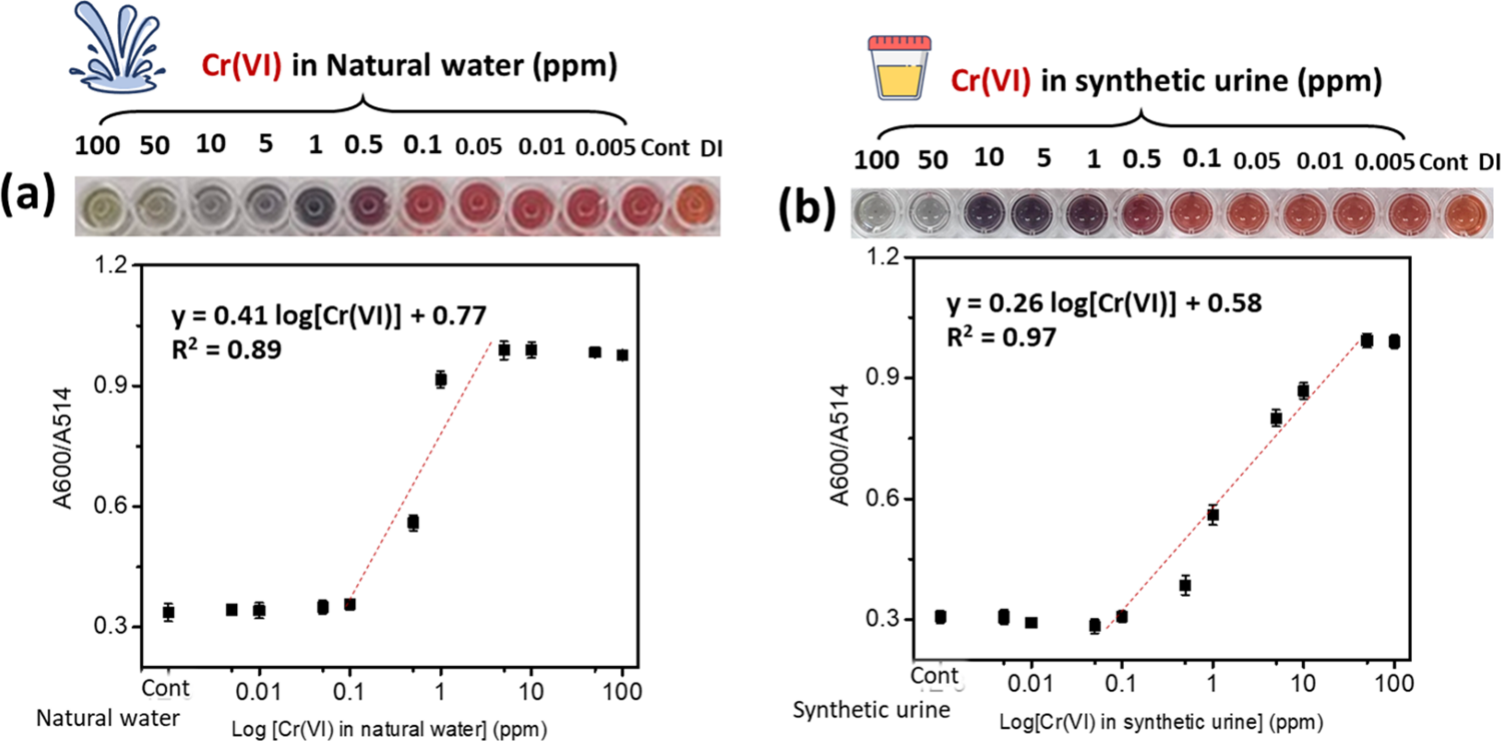
Sensitivity plot of the absorbance ratio at wavelengths of 600 and 517 nm (*A*_600 nm_/*A*_517 nm_) with respect to the concentration of Cr(VI) spiked in (a) natural water and (b) synthetic urine. The dashed straight lines are obtained by the least-squares method.

Because of the widespread distribution of Cr in the environment, trace amounts of this element may potentially contaminate soil, natural water, food, and beverages, presenting a toxic risk to consumers. This study aimed to assess the feasibility of determining chromium levels in various real samples, such as natural water and synthetic urine, using our colorimetric probe colloid. Natural water samples collected at the river in Chonburi Province, Thailand, were chosen as representative real samples. Synthetic urine was chosen as a potential representative sample because Cr(VI) can possibly be found in urine.^40^ As-received natural water samples were filtered, and synthetic urine was diluted 100-fold with DI water before use. Different concentrations of Cr(VI) ions ranging from 0.05 to 100 ppm were prepared by spiking a standard solution in each sample. The volume ratio of Na-AuNPs/cit-AgNPs was fixed at 3:1 (final volume = 100 μL), and the volume of Cr(VI) was fixed at 70 μL. The incubation time was increased to 1 h to obviously observe the color difference after the reaction. The spectral and color transformation responses were monitored by collecting UV–vis spectra and images. Figure 6a,b displays the sensitivity plot of the absorption ratio (*A*_600_/*A*_517_) against the logarithm of the Cr(VI) concentration spike in natural water and diluted synthetic urine, respectively. The inset of Figure 6 and Figure S12 display the corresponding images and extinction spectra of the probe colloid at various concentrations of Cr(VI) in natural water and synthetic urine, respectively. It was found that the color transformation profile of the probe colloid toward Cr(VI) detection in both samples was similar. The cutoff level at 0.5 ppm by visual detection was found for both media systems. Taking into account the sensitivity response illustrated in Figure 6, a wider linear detection range of Cr(VI) was found in synthetic urine (0.05–50 ppm) compared to that in natural water (0.1–5 ppm). The calculated LOD values were approximately about 0.039 and 0.019 ppm for Cr(VI) determination in natural water and synthetic urine, respectively. These findings underscore the utility of our colorimetric probe colloid for detecting Cr(VI) contamination in complex media solutions.

## 4 Conclusions

A simple metal NP-based colorimetric sensor for the selective sensing of Cr(VI) using a mixture of Na-AuNPs and cit-AgNPs was employed. The type of capping agent is a crucial factor in the fabrication of colorimetric sensors based on metallic nanoparticles. The combination of NaBH_4_-capped AuNPs and sodium citrate-capped AgNPs emerged as the optimal for the probe mixture, exhibiting excellent stability and sensing performance in the detection of Cr(VI) in TW. The probable mechanism underlying the detection of Cr(VI) ions in TW could entail redox etching between AuNPs or AgNPs by Cr ions, followed by the subsequent redeposition of Au^3+^ and Ag^+^ ions, leading to particle aggregation. The original color of Na-AuNPs and cit-AgNPs probe colloids was orange. The probe color gradually changed to dark reddish–purple, dark purplish–red, dark blue–violet, and gray when reacted with Cr(VI) in TW at concentrations of 0.05–0.1, 0.5–1, 5–10, and 50–100 ppm, respectively. The color change was induced by the morphological transformation of both AuNPs and AgNPs when reacted with Cr(VI)-contaminated TW, which can be monitored by UV–vis measurement. The linear relationship plot of the absorption ratio (*A*_600_/*A*_517_) against the concentration of Cr(VI) in TW was in the range of 0.05–50 ppm. The calculated LOD was 22.9 ppb, and the cutoff level by visual observation was found at about 50 ppb. This probe colloid showed good specificity toward Cr(VI) sensing. This probe colloid was applicable to detect Cr(VI) contamination in other real samples, including natural water and synthetic urine. Thanks to the synergistic effect of the Na-AuNPs/cit-AgNPs combination in enhancing the selectivity and stability of the probe in TW, this probe colloid could emerge as an alternative metallic NP-based colorimetric sensor toward Cr(VI) contamination in samples containing complex components.

## Supporting Information Available

Protocol of Na-AuNPs and cit-AgNPs synthesis; optimization of the Na-AuNPs/cit-AgNPs combination for Cr(VI) ion detection in TW; extinction spectra and digital image of the probe colloid (mixed Na-AuNPs and cit-AgNPs) in the presence and absence of Cr(VI) in TW; electrochemical measurement on bare SPCE; extinction spectra of the selectivity study, stability, and feasibility test in natural water and synthetic urine; summary of previous reports using metallic NPs toward Cr(VI) detection (PDF)
