# Supplementary material for: Colorimetric Sensor for Cr(VI) Ion Detection in Tap Water Using a Combination of AuNPs and AgNPs
Source: ACS Omega. 2024 Jun 6;9(24):26472–83. doi: 10.1021/acsomega.4c02699 (PMC11191111; doi:10.1021/acsomega.4c02699)
Supplement: Supplementary file 1 — ao4c02699_si_001.pdf [file ao4c02699_si_001.pdf]

Supporting Information

Colorimetric sensor for Cr(VI) ion detection in tap water using the combination of AuNPs and AgNPs

Kullavadee Karn-orachai[1]*, Panwadee Wattanasin[2], Aroonsri Ngamaroonchote[1]

[1]National Nanotechnology Center (NANOTEC), National Science and Technology Development Agency (NSTDA), Pathum Thani 12120, Thailand

[2]Faculty of Science, Center of Excellence for Innovation in Chemistry, Prince of Songkla University, Hat Yai, Thailand

* Corresponding author: e-mail address: kullavadee.kar@nanotec.or.th

Content

1. Details of Na-AuNPs synthesis.
2. Details of cit-AgNPs synthesis.
3. UV-Vis spectra and photographic image of each nanomaterial mixed with DI and TW.
4. Synthesis of Na-AuNPs at five different concentrations.
5. Optimization Na-AuNPs:cit-AgNPs combination for Cr(VI) ion detection in TW
6. Electrochemical measurement.
7. The relationship between particle size of both Na-AuNPs and cit-AgNPs upon addition of Cr(VI) solutions.
8. Extinction spectra of the probe for entire measurement range of Cr(VI) detection from 5 ppb to 100 ppm performed in tap water.
9. Extinction spectra of selectivity study.
10. Extinction spectra of the Na-AuNPs and cit-AgNPs upon storage
11. Extinction spectra of the probe for entire measurement range (5 ppb to 100 ppm) of Cr(VI) detection in natural water and synthetic urine.
12. Summary of other previous reports using metallic NPs towards Cr(VI) detection.

1. Details of AuNPs synthesis

In this study, AuNPs stabilized with two different capping agents ($NaBH_4$ and TSC) separately prepared. The $NaBH_4$ capped- and TSC capped-AuNPs were denoted as Na-AuNPs and cit-AuNPs, respectively. Na-AuNPs were synthesized via reduction reaction method using $NaBH_4$ as a reducing agent. Briefly, freshly prepared $NaBH_4$ solution in ice-cold water (0.1 M) was rapidly added in 0.25 mM $HAuCl_4$ aqueous solution (20 mL). The solution was stirring for 10 min to complete the reduction reaction. Moreover, The AuNPs capped with TSC were synthesized with a similar protocol to previous method with additional of TSC. To do this, 5mg TSC was added to $HAuCl_4$ aqueous solution followed by adding $NaBH_4$ solution. Both Au colloids were left for overnight at room temperature before use.

Particle size of Na-AuNPs can be altered by varying volume of gold precursor. In this study, four different sizes of AuNPs were prepared and the volume and concentration of each reagent were listed in **Table S1**.

Table S1 Volume of each reagent for AuNPs synthesis

**Diameter of AuNPs (nm)**	**20 mM $HAuCl_4$ (mL)**	**DI (mL)**	**0.1M $NaBH_4$ (mL)**	**Solution color**
3.91	0.25	19.75	0.6	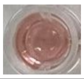
5.93	0.5	19.5	0.6	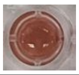
10.81	0.6	19.4	0.6	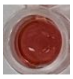
35.92	0.75	19.25	0.6	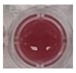
52.84	1	19	0.6	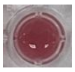

2. Details of cit-AgNPs synthesis

In this study, AgNPs stabilized with two different capping agents (NaBH4 and TSC) were prepared. The NaBH4 capped- and TSC capped -AgNPs were denoted as Na-AgNPs and cit-AgNPs, respectively. For Na-AgNPs synthesis, as-prepared NaBH4 aqueous solution (0.1 M, 0.3 mL) was rapidly injected in 0.3 mM AgNO3 aqueous solution (10 mL). To complete the reaction, the mixture was continuously stirred at 700 rpm for 10 min. The solution color changed from brown to light yellow. In case of cit-AgNPs synthesis, cit-AgNPs were prepared by reducing silver salt with TSC, which acts as both reducing and capping agents. Briefly, 0.368 mL of 10 mM AgNO3 aqueous solution was mixed with 23.75 mL DI water. Then, the solution was boiled (100 °C) and stirred at 700 rpm for 10 min. Freshly

prepared 1.25 mL of 1% TSC aqueous solution was quickly injected. The color was developed from clear to light yellow after continuously boiling for 30 min, confirming the formation of AgNPs. The colloidal solution of both Na-AgNPs and cit-AgNPs was stored in refrigerator before used.

Moreover, the particle size of Na-AgNPs can be tuned by varying volume of silver precursor. The volume and concentration of each reagent were listed in Table S2.

Table S2 Volume of each reagent for cit-AgNPs synthesis

**Particle size of cit-AgNPs (nm)**	**$AgNO_3$ (10 mM)**	**TSC (1%) (mL)**	**DI (mL)**	**Solution color**
37.05	0.276 mL	1.25 ml	23.75 ml	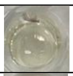
40.43	0.368 mL	1.25 ml	23.75 ml	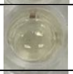
49.32	0.552 ml	1.25 ml	23.75 ml	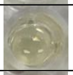
58.24	0.736 ml	1.25 ml	23.75 ml	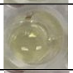
78.29	1.472 ml	1.25 ml	23.75 ml	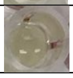

3. UV-Vis spectra and photographic image of each nanomaterial mixed with DI and TW.

The solid and dotted curves in Figure S1 are the extinction spectra of each metal colloid in the presence of DI water and tap water, respectively. The photograph image (inset on the right of Figure S1) displays the color transformation of each colloid after being mixed with DI and TW.

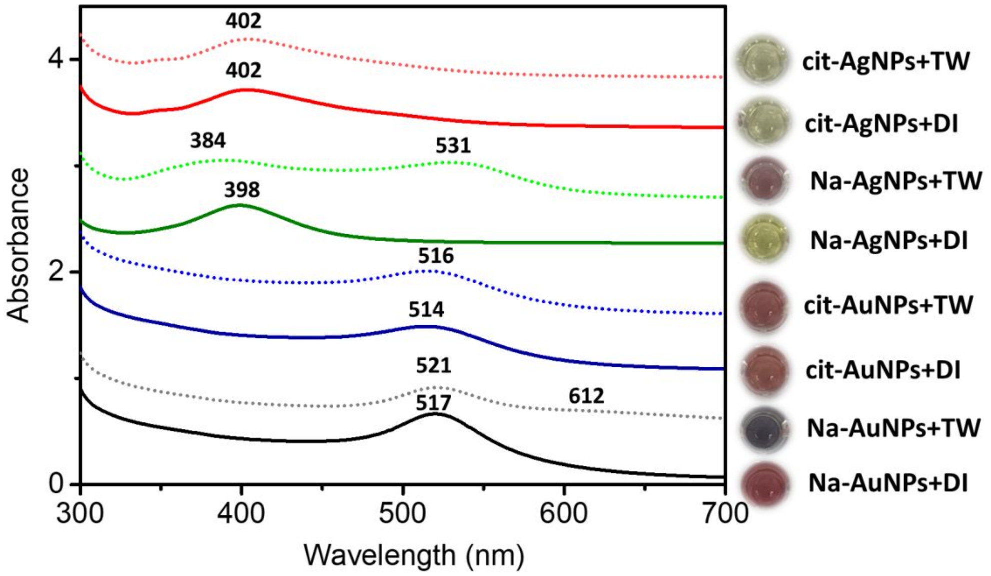

Figure S1 UV-Vis spectra of each nanomaterial mixed with DI and TW. Inset reveals the photographic image of resultant colloid upon DI and TW addition.

4. Synthesis of Na-AuNPs at five different concentrations

As the 5.93 nm-Na-AuNPs was selected as optimum particle, this size was quite small for centrifugation for variation of AuNPs concentration. The concentration of AuNPs was varied by varying the volume of all precursors as shown in **Table S3**. The concentration of Na-AuNPs colloid was observed by measuring optical density using spectrophotometer. **Fig. S2** exhibits the extinction spectra and inset (on the right) shows the corresponding photographic images of all Na-AuNPs at five different concentrations. These five concentrations of Na-AuNPs gave similar LSPR at the wavelength of 514 nm. The optical density (OD) varied from 0.30, 0.58, 0.87, 1.37, and 1.76. From photograph image, color intensity of probe colloid increased as increased concentration of Na-AuNPs.

Table S3 volume of each reagent for AuNPs synthesis during increase concentration of Na-AuNPs colloid.

OD of AuNPs (nm)	20 mM $HAuCl_4$ (mL)	DI (mL)	0.1M $NaBH_4$ (mL)
0.30	0.5	19.5	0.6
0.58	1	19	1.2
0.87	1.5	18.5	1.8
1.37	2	18	2.4
1.76	2.5	17.5	3.0

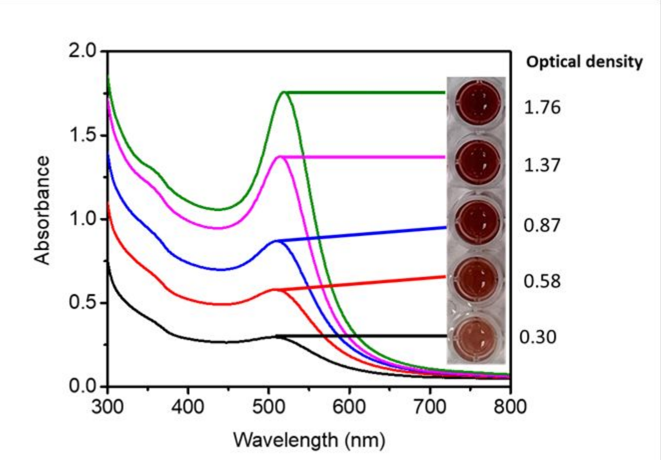

Figure S2 UV-Vis spectra of five different concentrations of Na-AuNPs (5.93 nm in diameter). Inset (on the right) shows the corresponding photograph taken under visible light.

5. Optimization Na-AuNPs:cit-AgNPs combination for Cr(VI) ion detection in TW

To achieve a suitable probe colloid for highly sensitive detection of Cr(VI) ion contaminated TW, the optimization of Na-AuNPs:cit-AgNPs combination including particle size of Na-AuNPs and cit-AgNPs, optical density of Na-AuNPs, ratio between Au:Ag, and volume of Cr(VI) ion in tap water was studied.

First, it is well known that particle size of metal NPs strongly influence the aggregation behavior of NPs in the solution, which directly affected the sensitivity and LOD of colorimetric probe. In this study, different particle sizes of Na-AuNPs (3.91, 5.93, 10.81, 35.92 and 52.84 nm) were synthesized by altering the amount of gold precursor in the synthesis reaction. The particle size of cit-AgNPs was fixed at about 40 nm. The volume ratio between AuNPs:AgNPs was fixed at 3:1 (final volume = 100 µL) and the volume of Cr(VI) was fixed at 50 µL. The concentration of Cr(VI) ranged from 0.1 to 100 ppm. UV-Vis spectra and photographs depicting the AuNPs:AgNPs combination upon addition of Cr(VI) are displayed in **Figure S3**. All AuNPs:AgNPs combinations with various sizes of Na-AuNPs show no aggregation upon addition of Cr(VI) ion. The efficacy of Cr(VI) detection varied with the particle sizes of Na-AuNPs. The probe combination of Na-AuNPs (3.91 and 5.93 nm):cit-AgNPs exhibited a highly sensitive response towards Cr(VI) determination as observed multicolor transformation from orange to purple, blue and finally green color. Nonetheless, the probe containing the smallest Na-AuNPs showed a pale color, which was hardly distinguished the correct color by naked eyes. The corresponding UV-Vis spectra of both systems (**Figure S3a** and **S3b**) reveal distinct spectral changes of Na-AuNPs:cit-AgNPs combination in the presence of Cr(VI) over entire measurement range. The pristine absorption bands of both cit-AgNPs and Na-AuNPs were observed at approximately 400 and 514 nm, respectively. The absorption band of AuNPs was gradually broadened with increasing the concentration of Cr(VI) ion. The characteristic absorption band of cit-AgNPs (400 nm) disappeared upon reaction with high Cr(VI) (5-100 ppm). The spectral response of LSPR of both Na-AuNPs and cit-AgNPs show the possible relationship for quantitative determination of Cr(VI) ion. In case of probe containing Na-AuNPs (10.81nm) and cit-AgNPs, the probe color changed to grey and dark grey upon mixing with Cr(VI) solution at concentrations of 10-100 and 0.5-5 ppm, respectively (**Figure S3c**). For larger Na-AuNPs (35.92 and 52.84 nm) mixed with cit-AgNPs, the color of probe solution notably changed at higher Cr(VI) content (10-100 ppm) (**Figure S3d** and **S3e**). No difference in color transformation by visual observation and less reduction of absorbance were observed when probe colloids reacted with lower concentration of Cr(VI) (0 to 5 ppm). These results indicate that different particle sizes of Na-AuNPs exhibit varying sensing behaviors in response to changes in Cr(VI) concentration. The mixed Na-AuNPs (5.93 nm) and cit-AgNPs (40 nm) showed the best response toward Cr(VI) detection. Consequently, Na-AuNPs (5.93 nm) was selected for further investigation.

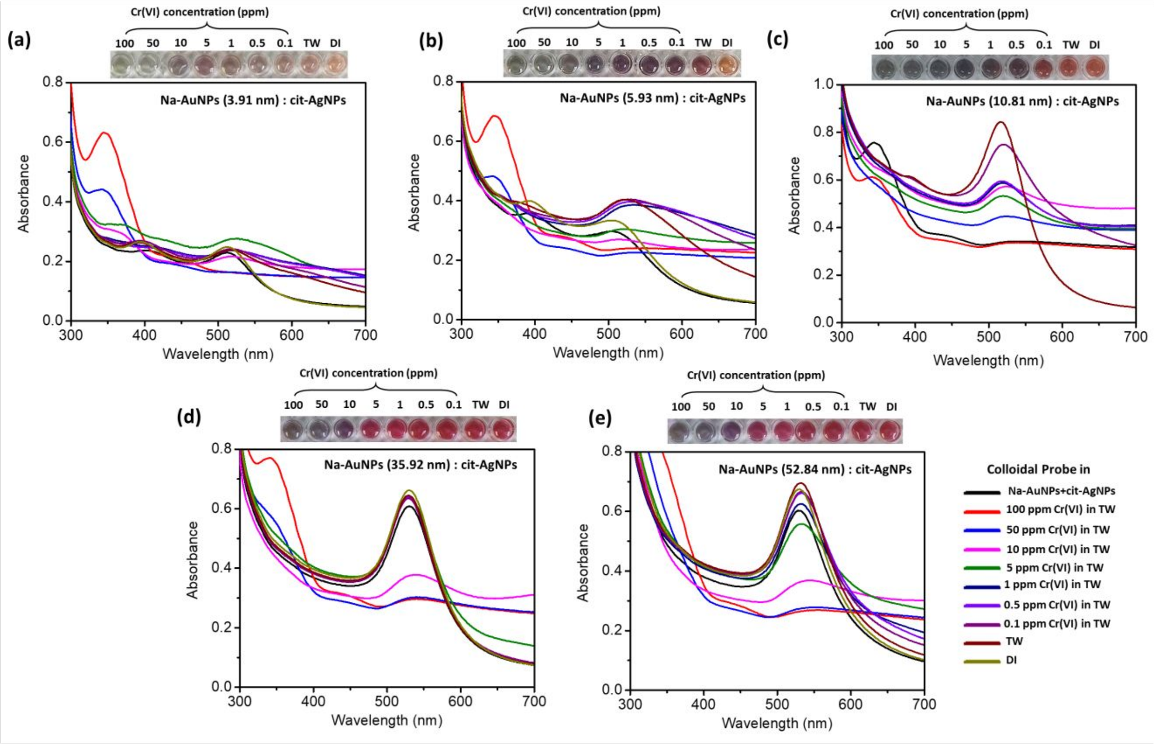

Figure S3 Extinction spectra and inset (on the top) shows the corresponding photograph image of probe colloid (Na-AuNPs:cit-AgNPs) upon Cr(VI) addition**.** Cit-AgNPs (40 nm in diameter) were separately mixed with five different particle size of Na-AuNPs including (a) 3.91, (b) 5.93, (c) 10.81, (d) 35.92 and (e) 52.84 nm-Na-AuNPs.

Subsequently, the particle size of cit-AgNPs was also varied (37.05, 40.43, 49.32, 58.24, and 78.29 nm) by changing silver precursor in the synthesis reaction. The 5.93-nm-Na-AuNPs were chosen to mix with cit-AgNPs to form probe colloid. The AuNPs:AgNPs:Cr volume ratio was kept at 3:1:2 (with a final volume of 150 µL). The concentration of Cr(VI) in tap water ranged from 0.1 to 100 ppm. The behavior of probe during Cr(VI) detection was monitored by collecting UV-Vis spectra and photographing the solution. **Figure S4** depicts the UV-Vis spectra and photograph image of Au:Ag probe solution using various particle sizes of cit-AgNPs upon Cr(VI) addition. By considering the photograph of all probe solutions, multicolor evolution from original orange to old-rose, dark purple, dark blue, and finally grey color was observed for all sizes of cit-AgNPs with different detection range. It was determined that broader detection range (0.5 to 100 ppm) was achieved when using cit-AgNPs with sizes of 37.05, 40.43, and 49.32 nm (**Figure S4a-S4c**). While, larger cit-AgNPs (58.24, and 78.29 nm) gave a narrow detection range at Cr(VI) concentration of 0.5 to 10 ppm (**Figure S4d-S4e**). The LSPR responses of all detection probe containing various sizes of cit-AgNPs gave similar pattern by observing broadened Na-AuNPs and reducing of cit-AgNPs LSPR bands. The probe system containing cit-AgNPs at sizes of 37.05, 40.43, and 49.32 nm showed the relationship of AuNPs absorbance peak variation upon Cr(VI) addition, suggesting potential utility for the quantitative determination of Cr(VI). Consequently, the 40.43-nm cit-AgNPs were selected as the optimal size for further investigation.

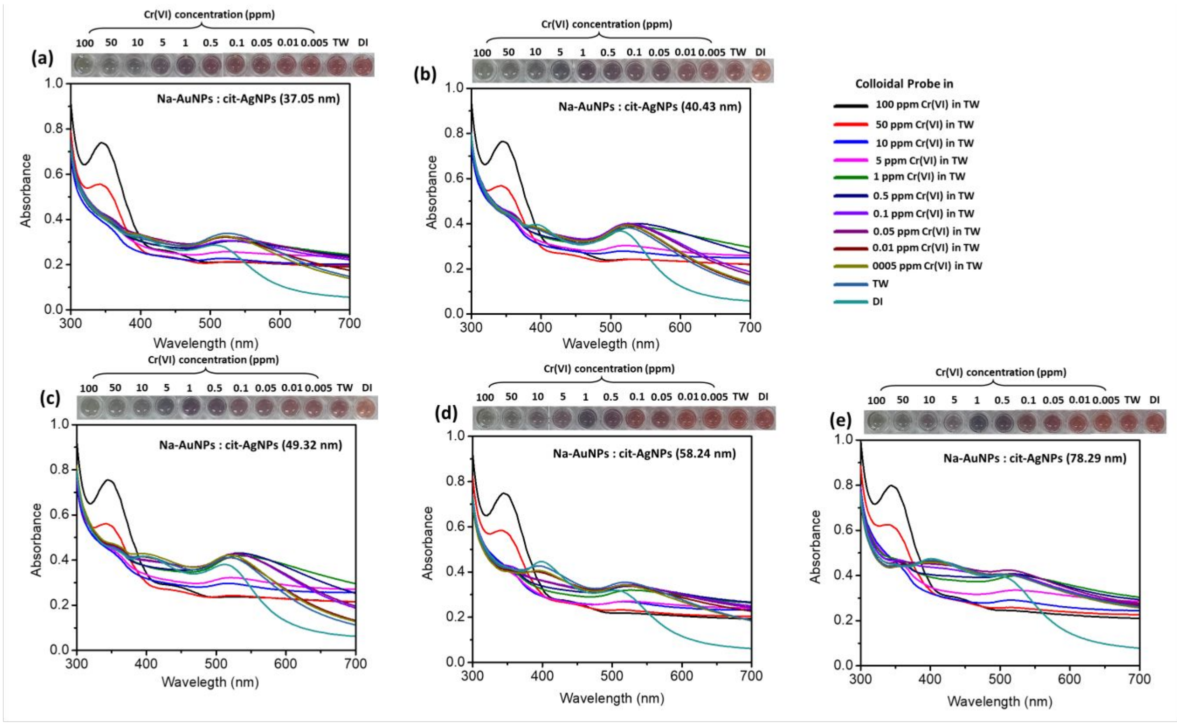

Figure S4 Extinction spectra and inset (on the top) shows the corresponding photograph image of probe colloid (Na-AuNPs:cit-AgNPs) upon Cr(VI) addition**.** Na-AuNPs (5.93 nm in diameter) were separately mixed with five different particle size of cit-AgNPs including (a) 37.05, (b) 40.43, (c) 49.32, (d) 58.24 and (e) 78.29 nm-cit-AgNPs.

Upon achieving the appropriate sizes of both Na-AuNPs (5.93 nm) and cit-AgNPs (40.43 nm), these particles demonstrated a wide detection range for Cr(VI) detection. However, this was hard to distinguish the color shade by naked eyes because of light-colored probe colloid. Enhancing the detection limit for naked-eye observation is crucial. It is assumed that the color evolution of this probe colloid mainly comes from the morphological change of AuNPs, which directly affected the alteration of absorption behavior of particles. Therefore, intense probe color was developed by increasing the concentration of Na-AuNPs. The concentration of Na-AuNPs was adjusted by increasing both gold precursor and reducing agent in the synthesis reaction. Detailed information regarding the synthesis protocol and characterization of AuNP concentration variation was provided in **Table S3** and **FigureS2**. The concentration of as-synthesized Na-AuNPs was monitored from optical density (OD) of Au colloid obtained from UV-Vis spectra. The OD value varied from 0.30 to 1.76. The Cr(VI) monitoring system was prepared by fixing the volume ratio of AuNPs:AgNPs:Cr at 3:1:2 (final volume = 150 μL). Cr(VI) spiked in TW was prepared in the concentration range of 0.01-100 ppm. **Figure S5** represents the UV-Vis spectra and photographic images of Au:Ag probe solution at different concentrations of Na-AuNPs upon Cr(VI) addition. From photograph, color intensity of probe colloid increased as increased concentration of Na-AuNPs. For LSPR response of probe colloid upon Cr(VI) addition, the variation of absorption band of Na-AuNPs was found when varied the concentration of Na-AuNPs. The wide

absorption peak (450-700 nm) was observed for probe solution containing lower concentration of Na-AuNPs (OD = 0.30, 0.58, and 0.87) (**Figure S5a-c**). Whereas narrower extinction band of Na-AuNPs (450-630 nm) was found for probe solution containing higher concentration of Na-AuNPs (OD = 1.37, and 1.76) (**Figure S5d-e**). These different LSPR responses of Na-AuNPs are possibly caused by the Na-AuNPs content. At lower Na-AuNPs concentration (OD = 0.30, 0.58, and 0.87), Cr(VI) can effectively decrease the absorption band and induce the aggregation of Na-AuNPs as seen low and wide extinction band. At higher concentrations of Na-AuNPs (OD = 1.36, and 1.95), the added amount of Cr(VI) was less than the quantity of Na-AuNPs. Consequently, some Na-AuNPs reacted with Cr(VI), leaving excess Na-AuNPs behind, resulting in a slight reduction with a narrower absorption band of Na-AuNPs. Solely considering the spectral response, the probe colloid containing the highest concentration of Na-AuNPs (OD = 1.95) exhibited the greatest sensitivity towards Cr(VI) sensing, evidenced by the reduction of LSPR band at 514 nm. However, the color of the probe at different Cr(VI) concentrations was challenging to determine by naked-eye observation. Therefore, achieving a balance between visual observation and LSPR response, the probe colloid containing Na-AuNPs at OD of 0.87 was selected as an optimum Na-AuNPs concentration for further investigation. This is because the response of color transformation upon Cr(VI) addition was easily distinguished by naked eyes and the linear response can possibly be created from the plot between absorbance ratio at 600:517 (A_{600}/A_{517}) and Cr(VI) content.

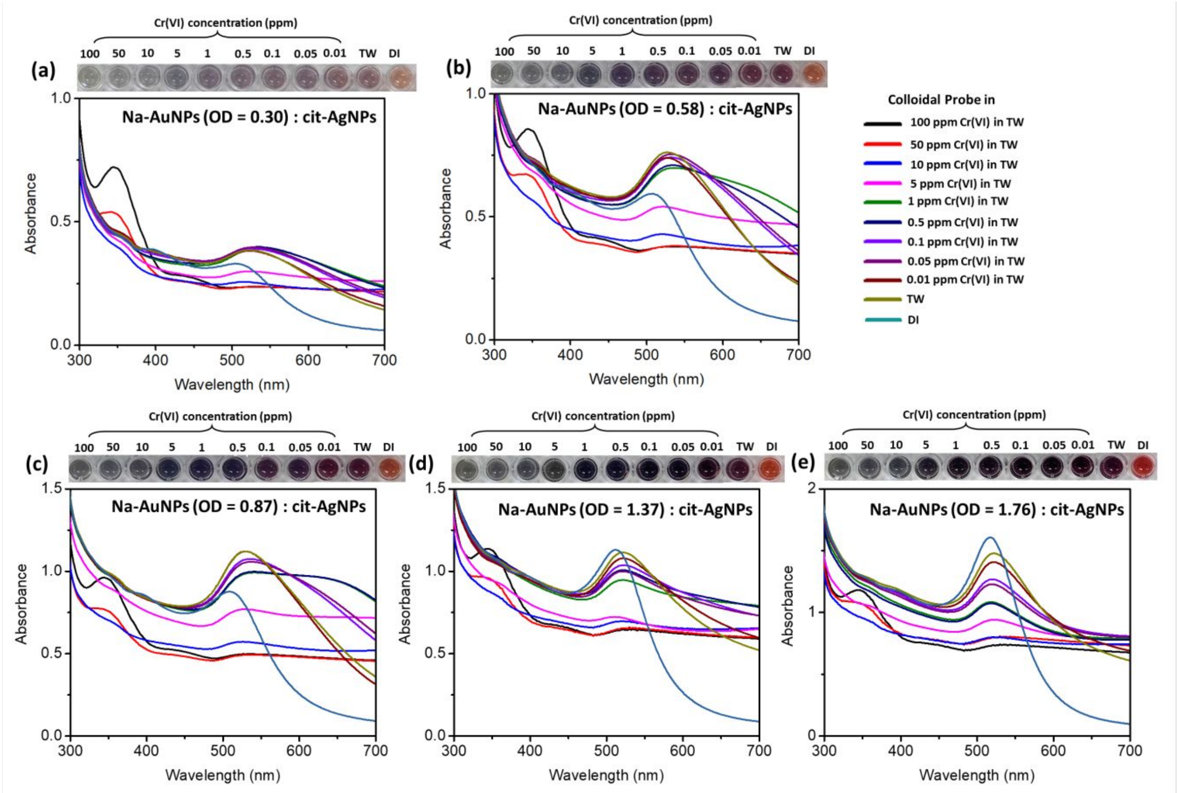

Figure S5 Extinction spectra and inset (on the top) shows the corresponding photograph image of probe colloid (Na-AuNPs:cit-AgNPs) upon Cr(VI) addition**.** Cit-AgNPs were separately mixed with five

different concentrations of Na-AuNPs (5.93 nm in diameter) by varying optical density at (a) 0.30, (b) 0.58, (c) 0.87, (d) 1.37 and (e) 1.76.

Moreover, the quantity of analyte is a critical factor influencing the color transformation and absorption spectra of the probe upon the addition of Cr(VI). In this investigation, the volume of Cr(VI) solution was varied between 25, 35, 50, 75 µL and added in probe colloid (with a fixed volume ratio of AuNPs:AgNPs to 3:1, totaling 100 µL). Both color change and optical property were monitored upon addition of Cr(VI) in the concentration range of 0.01 to 100 ppm. **Figure S6** shows the UV-Vis spectra and photographic image of Au:Ag probe solution at different volume of Cr(VI) addition. By considering the photograph image, detection system with different Cr(VI) volume gave different color evolution of the probe colloid. In case of small volume of Cr (VI) addition (**Figure S6a**), the probe color developed from orange to dark red, purple, blue, an grey color when reacted with high Cr(VI) concentration (0.5 to 100 ppm). This indicates that the small amount of analyte may not be sufficient to elicit a detectable color difference by naked eyes. For large volumes of Cr(VI) solution (50, 75 µL) (**Figure S6c and d**), the color of probe colloid changed to dark purple and grey color when mixed with Cr at concentrations ranging from 0 to 5 ppm and 10 to 100 ppm, respectively. This higher Cr(VI) amount can suddenly induce the aggregation of the probe for entire detection range. Therefore, this excess Cr content was not suitable for this developed colorimetric probe. When 35 µL of Cr(VI) solution was added to the probe colloid (**Figure S6b**), multicolor transformation from orange to dark red, purple, blue and finally grey-green color was found for Cr (VI) detection in the range of 0.05 to 100 ppm. Additionally, the LSPR responses among each Cr(VI) content in the detection probe were also investigated. Broadening of the Na-AuNPs and reduction of the cit-AgNPs were observed under all detection conditions. With increasing Cr(VI) volume, the absorption peak at longer wavelengths (> 600 nm) also increased. This is caused by the aggregated AuNPs induced by high content of Cr(VI). This result is in good agreement with the result of color development from photograph. According to the result, the suitable Cr(VI) volume of 35 µL showed a favorable system to visual detection process, which was chosen as an optimum Cr(VI) content for further investigation.

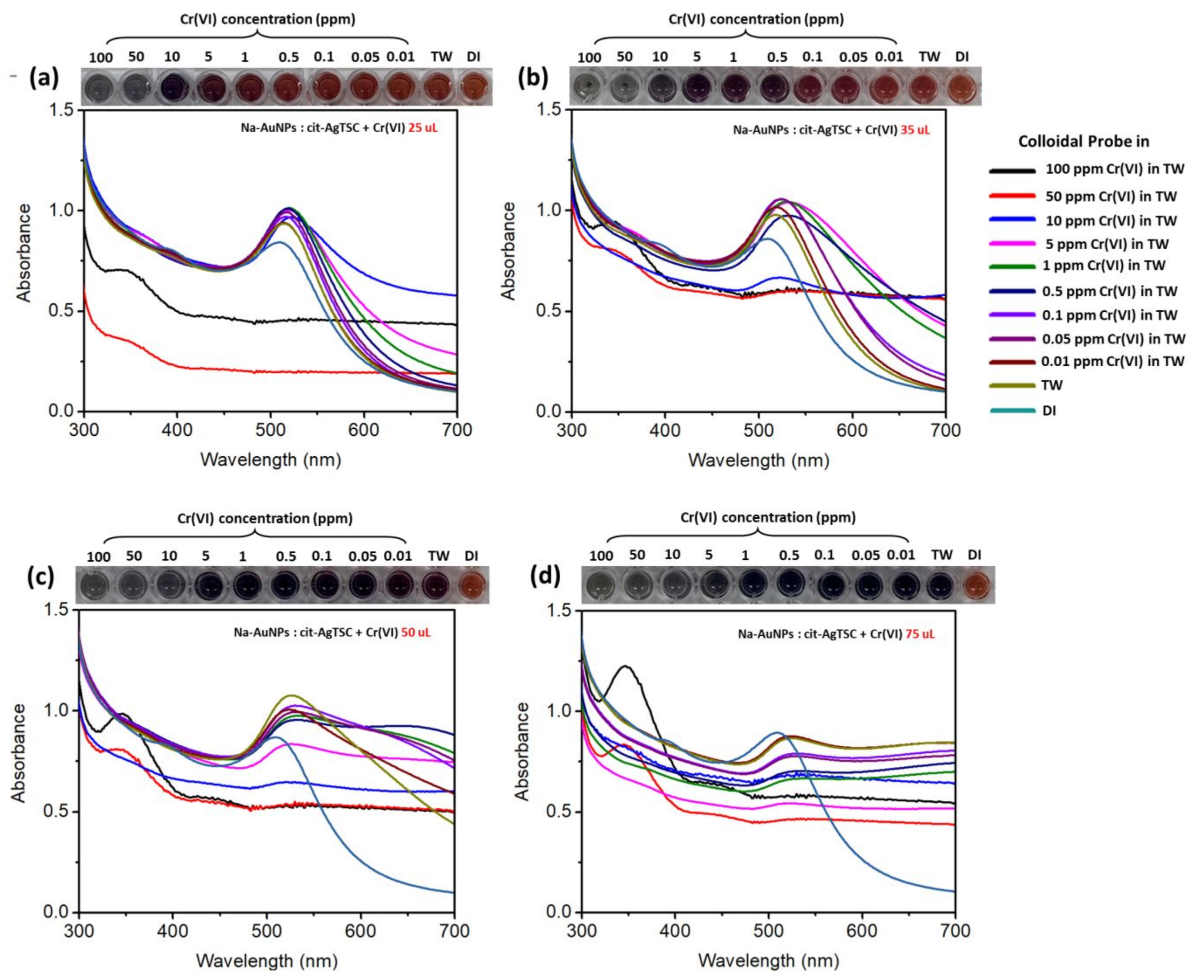

Figure S6 Extinction spectra and inset (on the top) shows the corresponding photograph image of probe colloid (Na-AuNPs:cit-AgNPs) toward Cr(VI) detection**.** The volume of Cr(VI) solution was varied from (a) 25, (b) 35, (c) 50, and (d) 75 µL.

Finally, determining the appropriate component ratio of the probe colloid, comprising Na-AuNPs and cit-AgNPs, emerges as a crucial parameter for achieving highly sensitive detection. The volume ratio between AuNPs and AgNPs varied from 9:1, 3:1, and 1:1 (resulting in a final volume of 100 µL). The standard Cr(VI) spiked in TW at concentration range of 0.01 to 100 ppm was used as analyte. Fixed volume of Cr(VI) solution (35 µL) was added in previous probe colloid. The detection performance was investigated by observing color evolution and UV-Vis spectra of the probe at different AuNPs:AgNPs ratios after reacting with different Cr(VI) concentrations. **Figure S7** illustrates the extinction spectra, with the inset showing the corresponding photographic image of the probe colloid at three different Au:Ag volume ratios upon the addition of Cr(VI). Each colorimetric probe system gave different spectral change and color transformation.It was found that the probe colloid containing high Na-AuNPs content (Au:Ag ratio = 9:1) exhibited the color change from original orange to dark purple

and grey color when mixed with Cr(VI) at concentrations of 0 to 5 ppm and 10 to 100 ppm, respectively. Upon the addition of Cr(VI) solution, a wide absorption band with a shoulder peak at longer wavelengths was observed for Na-AuNPs (**Figure S7a**). The presence of LSPR band at higher wavelength confirmed the formation of Na-AuNPs aggregation in the solution. The sensitivity of this probe colloid could not be enhanced by increasing the amount of Na-AuNPs. In the case of probe colloid with Au:Ag volume ratio of 1:1, the color of the probe obviously developed from orange to dark purple and grey when reacted with high Cr(VI) concentration of 5 to 100 ppm. While the color of probe in the control TW was comparable to probe reacted with Cr(VI) at concentration of 0 to 1 ppm. Moreover, two characteristic LSPR bands of both cit-AgNPs and Na-AuNPs were clearly observed at 400 and 514 nm, respectively (**Figure S7c**). The absorption band of both cit-AgNPs and Na-AuNPs gradually decreased with increasing Cr(VI) content (0 to 1 ppm). Subsequently, the LSPR peak vanished when exposed to high concentrations of Cr(VI) (5 to 100 ppm). This optical property gave a good agreement with the corresponding color transformation. This probe colloid gave a narrow visual detection range at high Cr(VI) content. For probe colloid prepared by mixing Na-AuNPs and cit-AgNPs at 3:1 volume ratio (**FigureS7b**), the multicolor evolution (original orange to reddish purple, dark purplish red, dark blue violet, and grey color) with wide visual detection range from 0 to 100 ppm Cr(VI) concentration was observed. The LSPR peak of Na-AuNPs gradually decreased with wide absorption wavelength as increased the Cr(VI) concentration (0 to 10 ppm). Subsequently, the characteristic LSPR bands of both AuNPs and AgNPs disappeared and addition absorption band at about 345 nm was found when reacted with high Cr(VI) concentration at 50 to 100 ppm. This probe system exhibited a correlation between the spectral changes in AuNPs bands upon the addition of Cr(VI), potentially enabling quantitative determination of Cr(VI). Furthermore, it achieved high sensitivity for Cr(VI) detection by visual observation. Considering all optimization results, the suitable detection system comprises (1) a probe colloid consisting of a mixture of 5.93-nm Na-AuNPs and 40.43-nm cit-AgNPs at a fixed volume ratio of AuNPs:AgNPs of 3:1, and (2) an optimum Cr(VI) volume of 35 μL. These parameters were employed for further analytical performance studies.

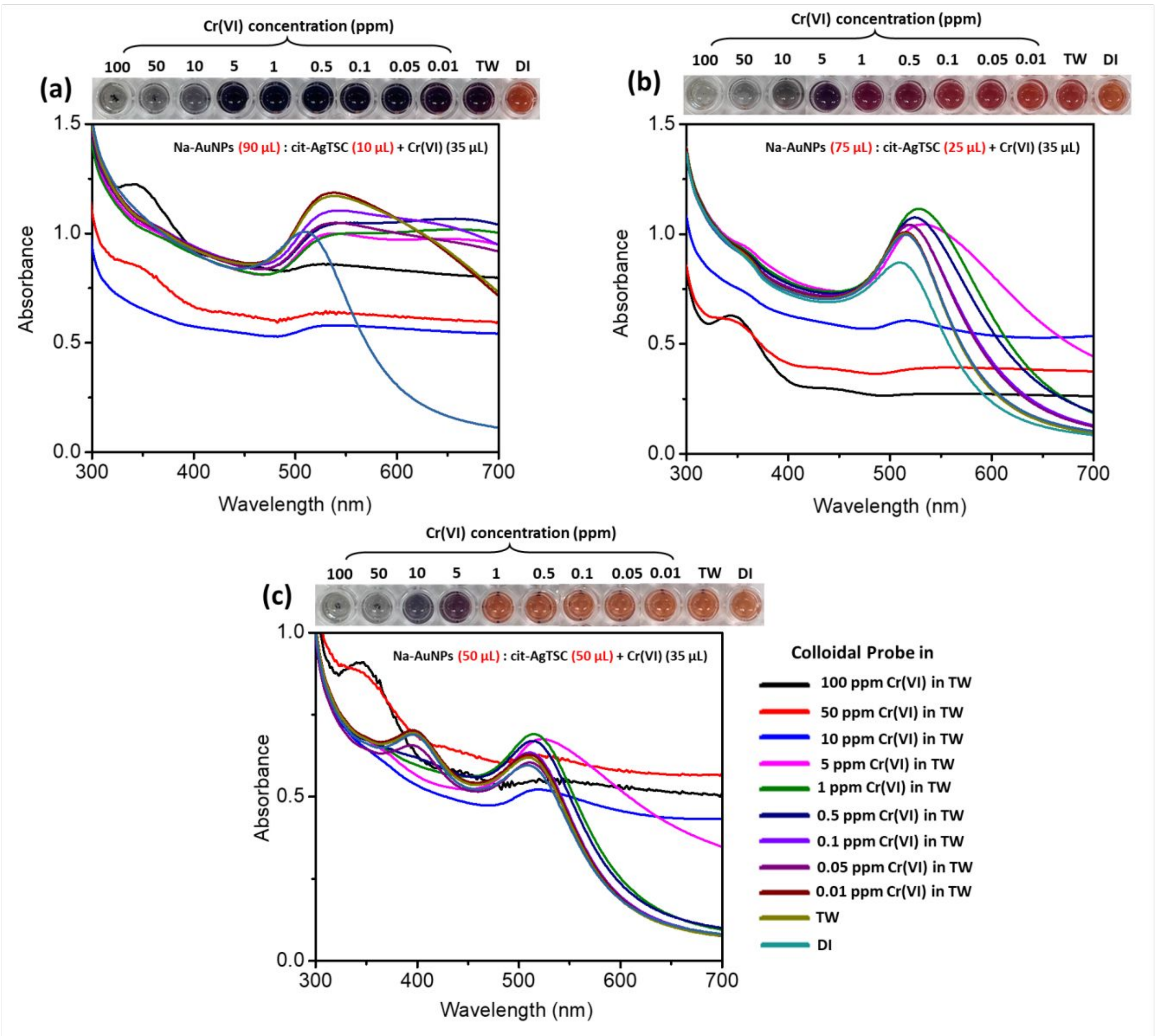

Figure S7 Extinction spectra and inset (on the top) shows the corresponding photograph image of probe colloid (Na-AuNPs:cit-AgNPs) at three different Au:Ag volume ratios upon Cr(VI) addition**.** The volume ration of Au:Ag was varied from (a) 9:1**,** (b) 3:1, and (c) 1:1.

6. Electrochemical measurement.

Electrochemical measurement was carried out using screen-printed carbon electrode consisting of carbon based working and auxiliary electrodes and Ag/AgCl reference electrode (SPCE, 100TE, Zensor) was selected as a disposable electrode. A 50 µL colloidal solution of the probe was applied directly to completely coat the electrode. Differential pulse voltammetry was recorded with portable potentiostat (electrochemical analyzer (ECAS 100), Zensor) incorporated with ECA100 software at scan rate of 50 $mV \cdot s^{-1}$. Differential pulse voltammograms were collected under ambient environment at room temperature.

7. The relationship between particle size of both Na-AuNPs and cit-AgNPs upon addition of Cr(VI) solutions.

The possible mechanism of our developed probe colloid was investigated by observing the shape evolution of both AuNPs and AgNPs upon addition of Cr(VI) ion in the solution. The correlation between the particle sizes of Na-AuNPs and cit-AgNPs upon the introduction of Cr(VI) solutions might provide insight into predicting the detection mechanism. Figure S8 shows the double y-axes plot, showing diameter of Na-AuNPs (on the left) and diameter of cit-AgNPs (on the right) versus the probe colloid reacted with different concentrations of Cr(VI) in TW.

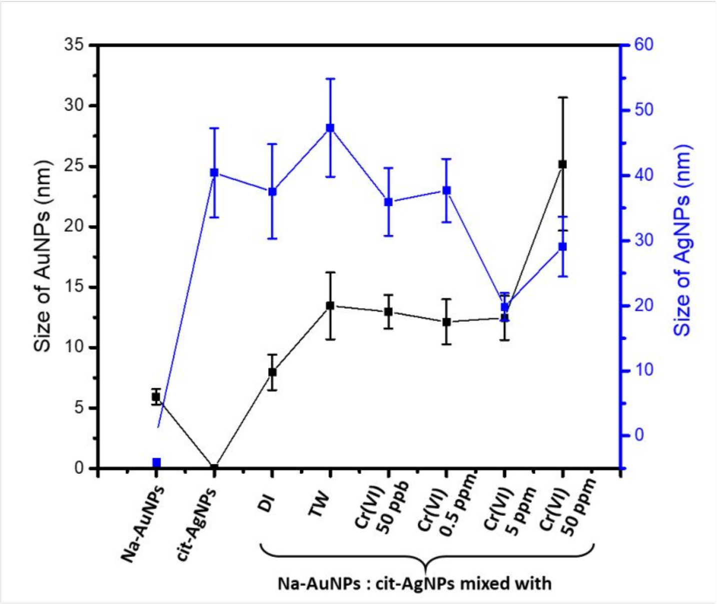

Figure S8 Plot with double y-axes: diameter of Na-AuNPs (on the left) and diameter of cit-AgNPs (on the right) versus the probe colloid reacted with different concentrations of Cr(VI) in TW.

8. **Extinction spectra of the probe for entire measurement range of Cr(VI) detection from 5 ppb to 100 ppm performed in tap water.**

Figure S9 depicts the spectral response of the probe colloid for entire measurement range of Cr(VI) detection (0 to 100 ppm). Tap water was used as a solvent in this study. The spectral shape changes by altering the amount of Cr(VI) content.

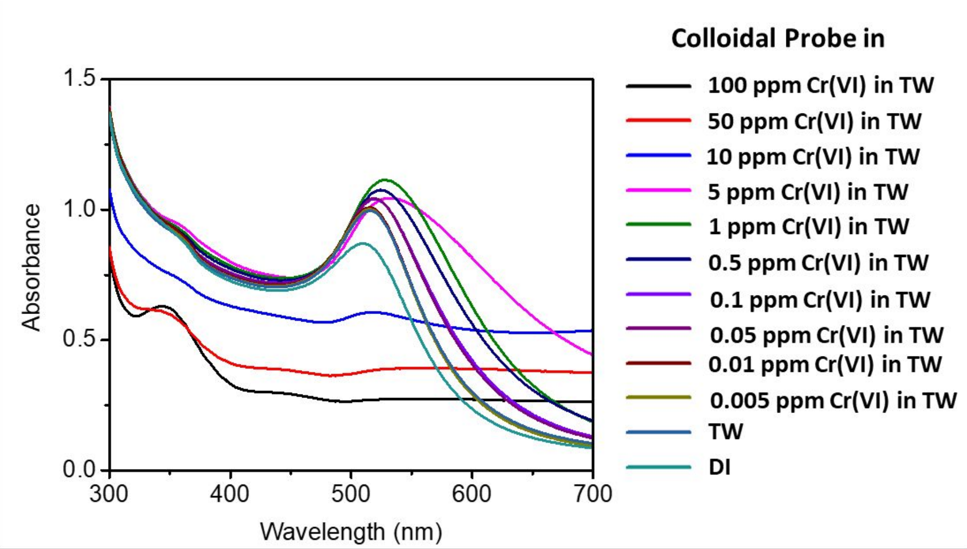

Figure S9 The corresponding extinction spectra of the probe for entire measurement range of Cr(VI) detection from 5 ppb to 100 ppm performed in tap water.

9. **Extinction spectra of selectivity study.**

Figure S10 shows the extinction spectra of probe colloid in the presence of various heavy metal ions (Pb, Cd, Ni Cu, Zn, Fe, Mn, Hg, As, Cr(III)) compared to Cr(VI) ion. A wider absorption band was only observed for probe colloid reacted with Cr(VI). Whereas extinction spectra of probe colloid reacted with other heavy metal ions gave similar result to the control sample of tap water.

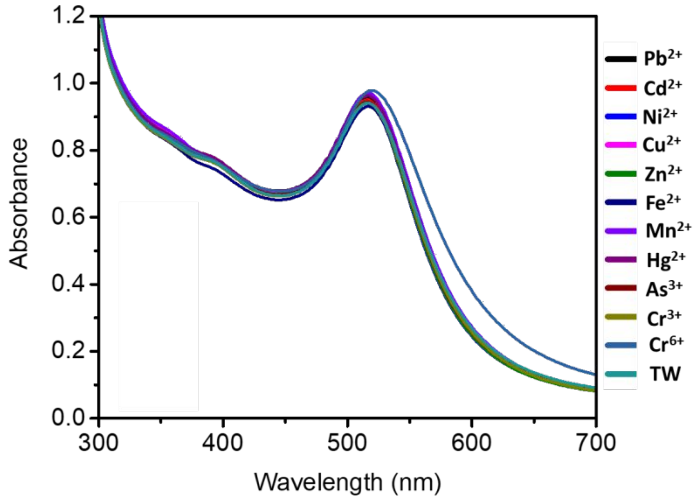

Figure S10 The corresponding extinction spectra of selectivity study. The concentration of all interfering ions was fixed at 0.1 ppm.

10. Extinction spectra of the Na-AuNPs and cit-AgNPs upon storage

The stability of individual Na-AuNPs and cit-AgNPs was separately investigated using UV-Vis measurement. Figure S11a and b indicate the extinction spectra of Na-AuNPs and cit-AgNPs upon 4 months storage, respectively, with photographic insets on the right side showing the resulting colloids. Both nanomaterials are stable upon storage time as observed no change of UV-Vis spectra and no change of solution color.

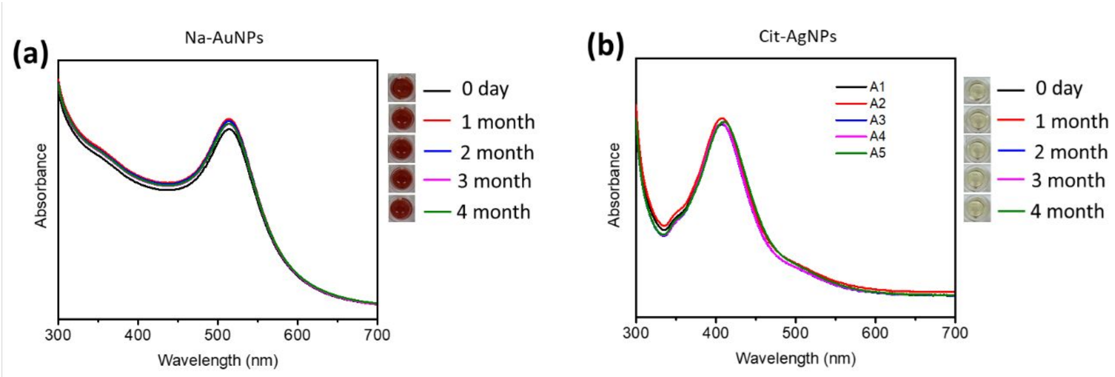

Figure S11 UV-Vis spectra of (a) Na-AuNPs and (b) cit-AgNPs upon 4 months storage. Inset reveals the photographic image of resultant colloid.

11. Extinction spectra of the probe for entire measurement range (5 ppb to 100 ppm) of Cr(VI) detection in natural water and synthetic urine.

The feasibility of determining chromium levels in various real samples, such as natural water and synthetic urine, using our developed colorimetric probe colloid was demonstrated. Figure S12a and b depict the spectral response of the probe colloid for entire measurement range of Cr(VI) detection (0 to 100 ppm) in natural water and synthetic urine, respectively.

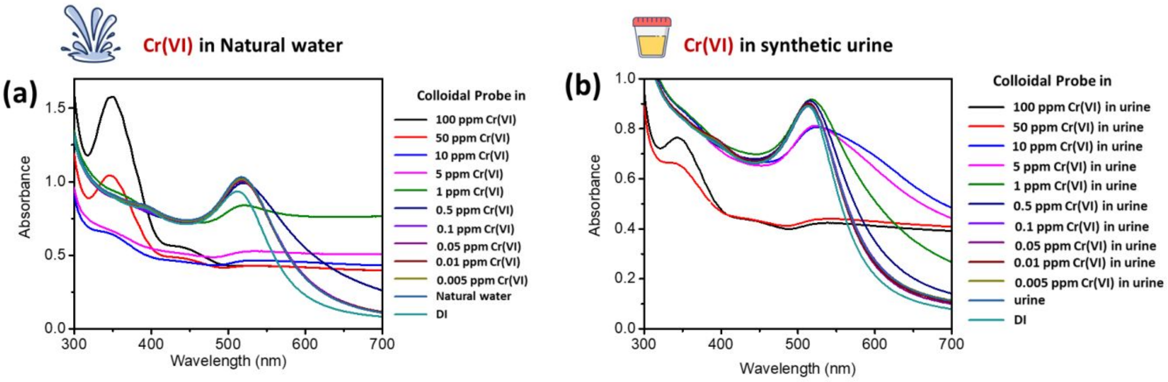

Figure S12 The corresponding extinction spectra of the probe for entire measurement range (5 ppb to 100 ppm) of Cr(VI) detection in (a) natural water and (b) synthetic urine.

12. Summary of other previous reports using metallic NPs towards Cr(VI) detection.

Table S3 Summary of other previous reports using different types and morphologies of metallic NPs towards Cr(VI) detection.

Metallic nanoparticles	LOD from spectrometer	LOD from naked eyes	Medium solution	Addition of other ions	References
Polyvinylpyrrolidone functionalized silver nanoparticles (PVP-AgNPs)	34 nM (1.75 ppb)	bright yellow to orange	PBS, pH 5.5	-	[1]
Maleic Acid-Functionalized Gold Nanoparticles	-	wine-red to gray color at1.0 ppb using smartphone	DI water	-	[2]
Starch-stabilized silver nanoparticles	0.93 µM (48 ppb)	-	Water, pH 3		[3]
Agcore–Aushell nanoparticles	0.01 µM (0.51 ppb)	0.1 µM (5.16 ppb)	DI water	Hexadecyl trimethyl ammonium bromide(CTAB)	[4]
gold nano-double cone @ silver nanorods	1.69 µM (87.23 ppb)	orange to pink, purple, finally colorless shade	DI water	Hydrogen bromide (HBr)	[5]
gold nanotetrapods (Au NTPs)	0.5 nM (0.025 ppb)	gray-green to blue-violet and pink color (3 nM) (0.15 ppb)	DI water	CTAB	[6]
Diglycolic acid-functionalized Au nanoparticles	0.32 ppb	Pink-red to deep blue	DI water	-	[7]
2-Mercapto-5-methyl-1,3,4-thiadiazole (MMT)-modified gold nanoparticles (MMT-AuNPs)	6.93 nM (0.346 ppb)	wine red to purple (12.4 nM) (from smartphone RGB color ratio)	DI water	-	[8]
graphene oxide (GO) nanoparticles	5.8 nM	transparent to blue	DI water	3,3,5,5-tetramethylbenzidine, H2O2 and 8-hydroxyquinoline (8-HQ)	[9]
Gold nanorods (GNRs)	88 nM	purple to red	Drinking water and sea water	pH 1.0 and 0.02 M CTAB, HCl	[10]

sodium hyaluronate functionalized gold nanoparticles (SH-AuNPs)	2.90 nM	wine red to blue gray (1 μM)	DI water	-	[11]
Sodium odium borohydride capped AuNPs: sodium citrate capped AgNPs mixture	22.9 ppb	50 ppb	Tap water	-	Our paper

References

1. S. He, X. Lin, H. Liang, F. Xiao, F. Li, C. Liu, P. Fan, S. Yang, Colorimetric detection of Cr(vi) using silver nanoparticles functionalized with PVP, Anal. Methods 11 (2019) 5819-5825.
2. A. Mohamed, X. Li, C. Li, X. Li, C. Yuan, H. Barakat, Smartphone-Based Colorimetric Detection of Chromium (VI) by Maleic Acid-Functionalized Gold Nanoparticles, Appl. Sci. 11 (2021) 10894.
3. W. Sapyen, S. Toonchue, N. Praphairaksit, A. Imyim, Selective colorimetric detection of Cr(VI) using starch-stabilized silver nanoparticles and application for chromium speciation, Spectrochim. Acta A Mol. Biomol. Spectrosc. 274 (2022) 121094.
4. J. Xin, F. Zhang, Y. Gao, Y. Feng, S. Chen, A. Wu, A rapid colorimetric detection method of trace Cr(VI) based on the redox etching of Ag core–Au shell nanoparticles at room temperature, Talanta, 101 (2012) 122-127.
5. S. Liu, X. Wang, C. Zou, J. Zhou, M. Yang, S. Zhang, D. Huo, C. Hou, Colorimetric detection of Cr6+ ions based on surface plasma resonance using the catalytic etching of gold nano-double cone @ silver nanorods, Anal. Chim. Acta 1149 (2021) 238141.
6. S. Wang, Y. Shi, H. Zhang, Y. Sun, F. Wang, L. Zeng, X. Li, A. Wu, Y. Zhang, Colorimetric sensor for Cr (VI) by oxidative etching of gold nanotetrapods at room temperature, Spectrochim. Acta A Mol. Biomol. Spectrosc. 295 (2023) 122589.
7. Y. Zhang, R. Bai, Z. Zhao, Q. Liao, P. Chen, W. Guo, C. Cai, F. Yang, Highly selective and sensitive probes for the detection of Cr(VI) in aqueous solutions using diglycolic acid-functionalized Au nanoparticle, RSC Adv., 2019, 9, 10958-10965.
8. R. Rajamanikandan, M. Ilanchelian, H. Ju, Smartphone-enabled colorimetric visual quantification of highly hazardous trivalent chromium ions in environmental waters and catalytic reduction of p-nitroaniline by thiol-functionalized gold nanoparticles, Chemosphere, 2023, 340, 139838.
9. N. N. Nghia, B. T. Huy, Y.-I. Lee, Colorimetric detection of chromium(VI) using graphene oxide nanoparticles acting as a peroxidase mimetic catalyst and 8-hydroxyquinoline as an inhibitor, Microchim Acta, 2019, 186, 36.
10. F.-M. Li, J.-M. Liu, X.-X. Wang, L.-P. Lin, W.-L. Cai, X. Lin, Y.-N. Zeng, Z.-M. Li, S.-Q. Lin, Non-aggregation based label free colorimetric sensor for the detection of Cr (VI) based on selective etching of gold nanorods, Sensors and Actuators B: Chemical, 2011, 155, 817-822
11. S. Li, T. Wei, G. Ren, F. Chai, H. Wu, F. Qu, Gold nanoparticles based colorimetric probe for Cr(III) and Cr(VI) detection, Colloids and Surfaces A: Physicochemical and Engineering Aspects, 2017, 535, 215-224
